# Allergic Rhinitis and Its Impact on Asthma (ARIA)‐EAACI Guidelines—2024–2025 Revision: Part II—Guidelines on Oral and Ocular Treatments

**DOI:** 10.1111/all.70305

**Published:** 2026-03-24

**Authors:** Rafael José Vieira, Bernardo Sousa‐Pinto, Jean Bousquet, Holger J. Schünemann, Torsten Zuberbier, Antonio Bognanni, Alkis Togias, Boleslaw Samolinski, Arunas Valiulis, Sian Williams, Anna Bedbrook, Wienczyslawa Czarlewski, Maria Jose Torres, Mohamed H. Shamji, Mário Morais‐Almeida, G. Walter Canonica, Leticia de las Vecillas, Mark S. Dykewicz, Cristina Jacomelli, Ludger Klimek, Lucas Leemann, Olga Lourenço, Nikolaos G. Papadopoulos, Ana Margarida Pereira, Marine Savouré, Sanna K. Toppila‐Salmi, Maria Teresa Ventura, Juan José Yepes‐Nuñez, Alvaro A. Cruz, Giorgio Ciprandi, Bilun Gemicioglu, Mattia Giovannini, Brigita Gradauskiene, Tuomas Jartti, Miloš Jeseňák, Piotr Kuna, Violeta Kvedariene, Désirée E. Larenas‐Linnemann, Amir H. A. Latiff, Yousser Mohammad, Ken Ohta, Padukudru A. Mahesh, Isabella Pali‐Schöll, Oliver Pfaar, Frederico S. Regateiro, Nicolas Roche, Luís Taborda‐Barata, Charlotte Suppli Ulrik, Giovanni Viegi, Luo Zhang, Tari Haahtela, Ivan Cherrez‐Ojeda, Juan Carlos Ivancevich, Nikolai Khaltaev, Arzu Yorgancioglu, Baharudin Abdullah, Mona Al‐Ahmad, Maryam Ali Al‐Nesf, Rita Amaral, Julijana Asllani, Karl‐C. Bergmann, Jonathan A. Bernstein, Michael S. Blaiss, Elina Toskala, Pedro Carreiro‐Martins, Thomas Casale, Lorenzo Cecchi, Alessandro Fiocchi, Antonio F. M. Giuliano, George Christoff, Ieva Cirule, Jaime Correia de Sousa, Elisio M. Costa, Philippe Devillier, Elham Hossny, Tomohisa Iinuma, Zhanat Ispayeva, Kaja Julge, Igor Kaidashev, Kazi S. Bennoor, Helga Kraxner, Inger Kull, Marek Kulus, Maciej Kupczyk, Andriy Kurchenko, Stefania La Grutta, Neven Miculinic, Lan Le Thi Tuyet, Sang Min Lee, Stephen Montefort, Andre Moreira, Joaquim Mullol, Rachel Nadif, Alla Nakonechna, Hugo E. Neffen, Marek Niedoszytko, Robyn E. O'Hehir, Ismail Ogulur, Yoshitaka Okamoto, Heidi Olze, Oscar Palomares, Petr Panzner, Vincenzo Patella, Constantinos Pitsios, Francesca Puggioni, Santiago Quirce, Agné Ramonaité, Maria Susana Repka‐Ramirez, Graham Roberts, Karla Robles‐Velasco, Menachem Rottem, Marianella Salapatas, Joaquin Sastre, Nicola Scichilone, Juan‐Carlos Sisul, Dirceu Solé, Manuel E. Soto‐Martinez, Milan Sova, Pongsakorn Tantilipikorn, Ana Todo‐Bom, Vladyslav Tsaryk, Ioanna Tsiligianni, Marilyn Urrutia‐Pereira, Erkka Valovirta, Tuula Vasankari, Dana Wallace, De Yun Wang, Margitta Worm, Osman M. Yusuf, Sara Gil‐Mata, Manuel Marques‐Cruz, Bassam Mahboub, Antonino Romano, Werner Aberer, Maria Cristina Artesani, Elena Azzolini, Bruno Barreto, Joan Bartra, Sven Becker, Bianca Beghe, Attilio Boner, Ewa Borowiack, Jacques Bouchard, Luisa Brussino, Roland Buhl, Francesco Catamerò, Denis Charpin, Niels H. Chavannes, Marta Chełmińska, Lei Cheng, Ekaterine Chkhartishvili, Seong H. Cho, Herberto Jose Chong‐Neto, Derek K. Chu, Cemal Cingi, Enrico Compalati, Raquel Albuquerque Costa, Biljana Cvetkovski, Victoria Cardona, Gennaro D'Amato, Janet M. Davies, Danilo Di Bona, Sandra N. Gonzalez Diaz, Maria V. Dimou, Maria Doulaptsi, Renato Ferreira‐da‐Silva, Radoslaw Gawlik, Mario Calvo‐Gil, Maximiliano R. Gómez, Maia Gotua, Christos Grigoreas, Maria Antonieta Guzman, Rachel House, Michael Hyland, Despo Ierodiakonou, Aspasia Karavelia, Paul Keith, Marta Kisiel, Tanja Soklic Kosak, Mitja Kosnik, Ilgim Vardaloglu Koyuncu, Vicky Kritikos, Justyna Litynska, Carlo Lombardi, Gilles Louis, Xin Luo, Matteo Martini, Cem Meço, Eris Mesonjesi, Florin Mihaltan, Marcin Moniuszko, Robert N. Naclerio, Sophia Neisinger, Markus Ollert, Michal Ordak, Giovanni Paoletti, Elena Parmelli, Edgar Arturo Perdomo‐Flores, Henrique Pereira, José Miguel Fuentes Pérez, Nhan Pham‐Thi, Emmanuel Prokopakis, Inês Ribeiro‐Vaz, Giovanni Rolla, Jan Romantowski, Philippe Rombaux, Philip W. Rouadi, Dermot Ryan, Ewelina Sadowska, Daiju Sakurai, Laila Salameh, Elie Serrano, Jane Da Silva, Michael B. Soyka, Krzysztof Specjalski, Vesna Tomic‐Spiric, Katarina Stevanovic, Abirami Subramaniam, Tuuli Thomander, Wu Tong, Martina Vachova, Marianne van Hage, Martin Wagenmann, Fanny Wai San Ko, Pascal Werminghaus, Paraskevi Vicky Xepapadaki, Yi‐Kui Xiang, Qintai Yang, He Zhang, Jaron Zuberbier, João A. Fonseca

**Affiliations:** ^1^ MEDCIDS—Department of Community Medicine, Information and Health Decision Sciences, Faculty of Medicine University of Porto Porto Portugal; ^2^ CINTESIS@RISE—Health Research Network, Faculty of Medicine University of Porto Porto Portugal; ^3^ Institute of Allergology, Charité—Universitätsmedizin Berlin, Corporate Member of Freie Universität Berlin and Humboldt‐Universität zu Berlin Berlin Germany; ^4^ Fraunhofer Institute for Translational Medicine and Pharmacology ITMP, Immunology and Allergology Berlin Germany; ^5^ ARIA (Allergic Rhinitis and Its Impact on Asthma) Montpellier France; ^6^ Department of Health Research Methods, Evidence, and Impact & Department of Medicine McMaster University Hamilton Ontario Canada; ^7^ Department of Biomedical Science Humanitas University, Pieve Emanuele Milan Italy; ^8^ Division of Allergy Immunology, and Transplantation (DAIT), National Institute of Allergy and Infectious Diseases, NIH Bethesda Maryland USA; ^9^ Department of Prevention of Environmental Hazards Allergology and Immunology, Medical University of Warsaw Warsaw Poland; ^10^ Institute of Clinical Medicine and Institute of Health Sciences Medical Faculty of Vilnius University Vilnius Lithuania; ^11^ Clinic of Asthma, Allergy and Chronic Lung Diseases Vilnius Lithuania; ^12^ International Primary Care Respiratory Group IPCRG Edinburgh UK; ^13^ Allergy Unit Regional University Hospital of Málaga, Malaga University, ARADyAL Malaga Spain; ^14^ National Heart and Lung Institute (NHLI), Imperial College London UK; ^15^ NIHR Imperial Biomedical Research Centre London UK; ^16^ Allergy Center CUF Descobertas Hospital Lisbon Portugal; ^17^ Personalized Medicine, Asthma and Allergy, IRCCS Humanitas Research Hospital, Rozzano Milano Italy; ^18^ Department of Allergy Hospital La Paz Institute for Health Research (IdiPAZ) Madrid Spain; ^19^ Section of Allergy and Immunology Saint Louis University School of Medicine Saint Louis Missouri USA; ^20^ “Respiriamo Insieme” Association, Asthma & Allergy Center Padova Italy; ^21^ Department of Otolaryngology Head & Neck Surgery, Universitätsmedizin Mainz Mainz Germany; ^22^ Allergology & Rhinology Department Center for Rhinology and Allergology Wiesbaden Germany; ^23^ Department of Political Science University of Zürich Zürich Switzerland; ^24^ RISE‐Health, Department of Medical Sciences, Faculty of Health Sciences University of Beira Interior Covilhã Portugal; ^25^ Allergy Department, 2nd Pediatric Clinic University of Athens Athens Greece; ^26^ Allergy Unit Instituto and Hospital CUF Porto Portugal; ^27^ Barcelona Institute for Global Health (ISGlobal) Barcelona Spain; ^28^ Universitat Pompeu Fabra (UPF) Barcelona Spain; ^29^ CIBER Epidemiología y Salud Pública (CIBERESP) Barcelona Spain; ^30^ Department of Allergy Skin and Allergy Hospital, Inflammation Center, Helsinki University Hospital and University of Helsinki Helsinki Finland; ^31^ Department of Otorhinolaryngology University of Eastern Finland and the North Savo Wellbeing Services County Kuopio Finland; ^32^ University of Bari Medical School Bari Italy; ^33^ Institute of Sciences of Food Production, National Research Council (ISPA‐CNR) Bari Italy; ^34^ School of Medicine Universidad de los Andes Bogotá DC Colombia; ^35^ Pulmonology Service, Internal Medicine Section Fundación Santa Fe de Bogotá, University Hospital Bogotá DC Colombia; ^36^ Fundacao ProAR and (Faculdade de Medicina da) Universidade Federal da Bahia Salvador Bahia Brazil; ^37^ Allergology Department Casa di Cura Villa Montallegro Genova Italy; ^38^ Department of Pulmonary Diseases Cerrahpaşa Faculty of Medicine, Istanbul University‐Cerrahpaşa Istanbul Turkey; ^39^ Department of Pulmonary Diseases Institute of Pulmonology and Tuberculosis, Istanbul University‐Cerrahpaşa Istanbul Turkey; ^40^ Department of Health Sciences University of Florence Florence Italy; ^41^ Allergy Unit Meyer Children's Hospital IRCCS Florence Italy; ^42^ Department of Immunology and Allergology Lithuanian University of Health Sciences Kaunas Lithuania; ^43^ Department of Pediatrics and Adolescent Medicine University of Turku, and Turku University Hospital Turku Finland; ^44^ Institute of Clinical Immunology and Medical Genetics, Department of Paediatrics and Adolescent Medicine, Department of Pulmonology and Phthisiology Jessenius Faculty of Medicine Martin Slovakia; ^45^ Comenius University Bratislava Slovakia; ^46^ University Hospital Martin Martin Slovakia; ^47^ Division of Internal Medicine Asthma and Allergy, Barlicki University Hospital, Medical University of Lodz Lodz Poland; ^48^ Institute of Clinical Medicine Clinic of Chest Diseases and Allergology, Faculty of Medicine, Vilnius University Vilnius Lithuania; ^49^ Institute of Biomedical Sciences, Department of Pathology, Faculty of Medicine Vilnius University Vilnius Lithuania; ^50^ Center of Excellence in Asthma and Allergy Médica Sur Clinical Foundation and Hospital México City Mexico; ^51^ Allergy & Immunology Centre Pantai Hospital Kuala Lumpur Kuala Lumpur Malaysia; ^52^ National Center for Research in Chronic Respiratory Diseases, Collaborating With WHO—EMRO Tishreen University School of Medicine Latakia Syria; ^53^ Pharmacy Department Al‐Sham Private University Damascus Syria; ^54^ Japan Antituberculosis Association (JATA), Fukujuji Hospital Tokyo Japan; ^55^ Department of Respiratory Medicine JSS Medical College, JSS Academy of Higher Education and Research Mysuru India; ^56^ Department of Biological Sciences and Pathophysiology University of Veterinary Medicine Vienna Austria; ^57^ Section of Rhinology and Allergy, Department of Otorhinolaryngology, Head and Neck Surgery University Hospital Marburg, Philipps‐Universität Marburg Marburg Germany; ^58^ Allergy and Clinical Immunology Department Hospitais da Universidade de Coimbra, Unidade Local de Saúde de Coimbra Coimbra Portugal; ^59^ Center for Innovative Biomedicine and Biotechnology (CIBB), Faculty of Medicine University of Coimbra Coimbra Portugal; ^60^ Institute of Immunology, Faculty of Medicine University of Coimbra Coimbra Portugal; ^61^ RISE‐Health, Faculty of Health Sciences, and UBIAir—Clinical & Experimental Lung Centre University of Beira Interior Covilhã Portugal; ^62^ Pneumologie AP‐HP Centre Université de Paris Cité, Hôpital Cochin Paris France; ^63^ UMR 1016, Institut Cochin Paris France; ^64^ Inserm, Equipe d'Epidémiologie Respiratoire Intégrative, CESP Villejuif France; ^65^ Department of Immunoallergology Unidade Local de Saúde, Cova da Beira Covilhã Portugal; ^66^ Department of Respiratory Medicine Copenhagen University Hospital‐Hvidovre Copenhagen Denmark; ^67^ Institute of Clinical Medicine, University of Copenhagen Copenhagen Denmark; ^68^ Pulmonary Environmental Epidemiology Unit CNR Institute of Clinical Physiology Pisa Italy; ^69^ Department of Otolaryngology‐Head and Neck Surgery The Third Affiliated Hospital of Sun Yat‐sen University Guangzhou China; ^70^ Skin and Allergy Hospital, Helsinki University Hospital, and University of Helsinki Helsinki Finland; ^71^ Universidad Espíritu Santo Samborondón Ecuador; ^72^ Department of Allergology & Pulmonology Respiralab Research Group Guayaquil Guayas Ecuador; ^73^ Servicio de Alergia e Immunologia, Clinica Santa Isabel Buenos Aires Argentina; ^74^ Global NCD Platform Geneva Switzerland; ^75^ Department of Pulmonary Diseases, Faculty of Medicine Celal Bayar University Manisa Turkey; ^76^ Department of Otorhinolaryngology—Head and Neck Surgery, School of Medical Sciences Universiti Sains Malaysia Kubang Kerian Kelantan Malaysia; ^77^ Microbiology Department, College of Medicine Kuwait University Kuwait City Kuwait; ^78^ Adult Allergy and Immunology Division—Hamad Medical Corporation Doha Qatar; ^79^ Department of Cardiovascular and Respiratory Sciences Porto Health School, Polytechnic Institute of Porto Porto Portugal; ^80^ Department of Women's and Children's Health Paediatric Research, Uppsala University Uppsala Sweden; ^81^ Department of Internal Medicine University of Medicine Tirana Albania; ^82^ Division of Immunology, Allergy and Rheumatology, Department of Medicine University of Cincinnati College of Medicine Cincinnati Ohio USA; ^83^ Department of Pediatrics Medical College of Georgia at Augusta University Augusta Georgia USA; ^84^ Department of Otolaryngology‐Head & Neck Surgery Thomas Jefferson University Philadelphia Pennsylvania USA; ^85^ NOVA Medical School/Comprehensive Health Research Centre (CHRC) Lisbon Portugal; ^86^ Serviço de Imunoalergologia, Centro Hospitalar de Lisboa Central/ULS São José Lisbon Portugal; ^87^ Division of Allergy/Immunology University of South Florida Tampa Florida USA; ^88^ Allergy and Clinical Immunology Unit San Giovanni di Dio Hospital Florence Italy; ^89^ Allergy Department Bambino Gesù Children's Hospital, IRCCS Rome Italy; ^90^ Clinica Medica “A. Murri”, Department of Preventive and Regenerative and Ionian Area (DiMePre‐J) University of Bari “Aldo Moro”, AOUC Policlinico Hospital Bari Italy; ^91^ Bulgarian Alliance for Clinical and Translational Allergy Sofia Bulgaria; ^92^ Latvian Association of Allergists, University Children Hospital Riga Latvia; ^93^ Life and Health Sciences Research Institute (ICVS), School of Medicine University of Minho Braga Portugal; ^94^ CINTESIS@RISE, Biochemistry Lab, Faculty of Pharmacy and Competence Center on Active and Healthy Ageing University of Porto Porto Portugal; ^95^ VIM Suresnes, UMR 0892, Pôle des Maladies des Voies Respiratoires, Hôpital Foch, Université Paris‐Saclay Suresnes France; ^96^ Pediatric Allergy, Immunology and Rheumatology Unit Children's Hospital, Ain Shams University Cairo Egypt; ^97^ Department of Otorhinolaryngology Chiba University Chiba Japan; ^98^ Department of Allergology and Clinical Immunology Kazakhstan Association of Allergology and Clinical Immunology, Kazakh National Medical University Almaty Kazakhstan; ^99^ Institute of Clinical Medicine, Children's Clinic, Tartu University Tartu Estonia; ^100^ Immunology & Allergology Department Poltava State Medical University Poltava Ukraine; ^101^ Department of Respiratory Medicine National Institute of Diseases of the Chest and Hospital Dhaka Bangladesh; ^102^ Department of Otorhinolaryngology Head and Neck Surgery, Semmelweis University Budapest Hungary; ^103^ Department of Clinical Science and Education Södersjukhuset, Karolinska Institutet Stockholm Sweden; ^104^ Sach's Children and Youth Hospital, Södersjukhuset Stockholm Sweden; ^105^ Department of Pediatric Respiratory Diseases and Allergology Medical University of Warsaw Warsaw Poland; ^106^ Department of Clinical and Laboratory Immunology Allergology and Medical Genetics, Bogomolets National Medical University Kyiv Ukraine; ^107^ Institute of Translational Pharmacology (IFT)‐National Research Council (CNR) Palermo Italy; ^108^ Croatian Pulmonary Society, Clinical Center for Pulmonary Diseases Zagreb Croatia; ^109^ Asthma, COPD Outpatient Care Unit University Medical Center Hô‐Chi‐Minh City Vietnam; ^110^ Division of Respiratory Disease and Allergy, Department of Internal Medicine Dankook University College of Medicine Cheonan Republic of Korea; ^111^ Department of Medicine, Faculty of Medicine and Surgery University of Malta Msida Malta; ^112^ EPIUnit‐Institute of Public Health, University of Porto, and Laboratory for Integrative and Translational Research in Population Health (ITR) Porto Portugal; ^113^ Serviço de Imunoalergologia, Centro Hospitalar Universitário São João Porto Portugal; ^114^ Basic and Clinical Immunology Unit, Department of Pathology, Faculty of Medicine University of Porto Porto Portugal; ^115^ Rhinology Unit & Smell Clinic, ENT Department Hospital Clínic Barcelona Spain; ^116^ Clinical & Experimental Respiratory Immunoallergy FRCB‐IDIBAPS, CIBERES, University of Barcelona Barcelona Spain; ^117^ Université Paris‐Saclay, UVSQ, Université Paris‐Sud Villejuif France; ^118^ Imperial College Healthcare NHS Trust London UK; ^119^ University of Liverpool Liverpool UK; ^120^ Center of Allergy, Immunology and Respiratory Diseases Santa Fe Argentina; ^121^ Department of Allergology Medical University of Gdansk Gdansk Poland; ^122^ Allergy, Asthma and Clinical Immunology, Alfred Health and Department of Immunology, School of Translational Medicine Monash University Melbourne Victoria Australia; ^123^ Swiss Institute of Allergy and Asthma Research (SIAF), University of Zurich Davos Switzerland; ^124^ ENT Department Chiba Rosai Hospital Chiba Japan; ^125^ Department of Otolaryngology, Head and Neck Surgery Chiba University Hospital Chiba Japan; ^126^ Department of Otorhinolaryngology Charité—Universitätsmedizin Berlin Berlin Germany; ^127^ Department of Biochemistry and Molecular Biology, School of Chemistry Complutense University of Madrid Madrid Spain; ^128^ Department of Immunology and Allergology, Faculty of Medicine in Pilsen Charles University Prague Czech Republic; ^129^ Division of Allergy and Clinical Immunology, Department of Medicine “Santa Maria della Speranza” Hospital Battipaglia Salerno Italy; ^130^ Division of Allergy and Clinical Immunology, Department of Medicine Agency of Health ASL Salerno Italy; ^131^ Postgraduate Programme in Allergy and Clinical Immunology, University of Naples Federico II Naples Italy; ^132^ Medical School University of Cyprus Nicosia Cyprus; ^133^ Department of Pulmonology and Allergology Klaipeda National Hospital Klaipeda Lithuania; ^134^ Vilnius University Medical Faculty Vilnius Lithuania; ^135^ Department of Allergy Clinics Hospital, National University San Lorenzo Paraguay; ^136^ Faculty of Medicine University of Southampton Southampton UK; ^137^ The David Hide Asthma and Allergy Centre St Mary's Hospital Isle of Wight UK; ^138^ NIHR Southampton Biomedical Research Centre, University Hospital Southampton NHS Foundation Trust Southampton UK; ^139^ LEADER Research Inc Hamilton Ontario Canada; ^140^ Division of Allergy, Asthma and Clinical Immunology Emek Medical Center Afula Israel; ^141^ Rappaport Faculty of Medicine Technion‐Israel Institute of Technology Haifa Israel; ^142^ ARIA, Asthma, Rhinitis, Immunology & Allergy Department Athens Greece; ^143^ Allergy Service, Fundacion Jimenez Diaz, Autonoma University of Madrid, CIBERES‐ISCIII Madrid Spain; ^144^ PROMISE Department University of Palermo Palermo Italy; ^145^ Allergy & Asthma, Medical Director, CLINICA SISUL, FACAAI, SPAAI Asuncion Paraguay; ^146^ Division of Allergy, Clinical Immunology and Rheumatology, Department of Pediatrics Federal University of São Paulo São Paulo Brazil; ^147^ Division of Respiratory Medicine, Department of Pediatrics Hospital Nacional de Niños, Universidad de Costa Rica San Jose Costa Rica; ^148^ Department of Respiratory Medicine and Tuberculosis University Hospital Brno Czech Republic; ^149^ Department of Otolaryngology Faculty of Medicine Siriraj Hospital, Mahidol University Bangkok Thailand; ^150^ Imunoalergologia, Centro Hospitalar Universitário de Coimbra, Faculty of Medicine, University of Coimbra Coimbra Portugal; ^151^ International Primary Care Respiratory Group IPCRG Aberdeen UK; ^152^ Health Planning Unit, Department of Social Medicine, Faculty of Medicine University of Crete Heraklion Greece; ^153^ Department of Medicine Federal University of Pampa Uruguaiana Brazil; ^154^ Department of Lung Diseases and Clinical Immunology University of Turku Turku Finland; ^155^ FiLHA, Finnish Lung Health Association Helsinki Finland; ^156^ Department of Clinical Medicine Pulmonary Diseases and Clinical Allergology, University of Turku Turku Finland; ^157^ College of Allopathic Medicine Nova Southeastern University Fort Lauderdale Florida USA; ^158^ Department of Otolaryngology, Yong Loo Lin School of Medicine National University of Singapore Singapore Republic of Singapore; ^159^ Division of Allergy and Immunology, Department of Dermatology, Allergy and Venerology Charité Universitätsmedizin Berlin Berlin Germany; ^160^ Allergy & Asthma Department The Allergy and Asthma Institute Islamabad Pakistan; ^161^ Pulmonary Department Rashid Hospital, DUBAI Health Dubai UAE; ^162^ Research Institute of Medical and Health Sciences, University of Sharjah Sharjah UAE; ^163^ Oasi Research Institute‐IRCCS Troina Italy; ^164^ BIOS S.p.A. Società Benefit Rome Italy; ^165^ Department of Dermatology Medical University of Graz Graz Austria; ^166^ Clinical Epidemiology and Research Center, Department of Biomedical Sciences Humanitas University Pieve Emanuele Milan Italy; ^167^ Department of Allergy and Immunology Para State University Center—CESUPA Belém Brazil; ^168^ Allergy Department Hospital Clinic and IDIBAPS, Universitat de Barcelona Barcelona Spain; ^169^ RICORS de Enfermedades Inflamatorias (ISCIII) Madrid Spain; ^170^ Department for Otorhinolaryngology Head and Neck Surgery, University of Tübingen Tübingen Germany; ^171^ Respiratory Medicine, Department of Maternal, Child and Adult Medical and Surgical Sciences University of Modena & Reggio Emilia, Azienda Ospedaliera‐Universitaria di Modena Modena Italy; ^172^ Pediatric Unit, Department of Surgical Sciences, Dentistry, Gynecology and Pediatrics University of Verona Verona Italy; ^173^ Evidence Prime Kraków Poland; ^174^ Evidence Prime Hamilton Ontario Canada; ^175^ Hôpital de la Malbaie (CIUSSS), Laval University Quebec City Quebec Canada; ^176^ Department of Medical Sciences University of Turin Turin Italy; ^177^ Allergy and Clinical Immunology Unit Mauriziano Hospital Torino Italy; ^178^ Department of Pulmonary Medicine Mainz University Hospital Mainz Germany; ^179^ Clinique des Bronches, Allergie et Sommeil Hôpital Nord Marseille France; ^180^ Department of Public Health and Primary Care Leiden University Medical Centre Leiden the Netherlands; ^181^ National eHealth Living Lab Leiden the Netherlands; ^182^ Department of Allergology and Otorhinolaryngology The First Affiliated Hospital, Nanjing Medical University Nanjing China; ^183^ Allergist, David Tvildiani Medical University Tbilisi Georgia; ^184^ College of Medicine, Division of Allergy‐Immunology, Department of Internal Medicine University of South Florida Tampa Florida USA; ^185^ Division of Allergy and Immunology Federal University of Parana Curitiba Brazil; ^186^ Evidence in Allergy Group, McMaster University and The Research Institute of St. Joe's Hamilton Hamilton Canada; ^187^ Medical Faculty, ENT Department Eskisehir Osmangazi University Eskisehir Turkey; ^188^ Scientific & Medical Department Lofarma S.p.A. Milan Italy; ^189^ Woolcock Institute of Medical Research Sydney Australia; ^190^ Department of Allergy Hospital Universitari Vall d'Hebron Barcelona Spain; ^191^ Division of Respiratory and Allergic Diseases High Specialty Hospital ‘A Cardarelli’, and Respiratory Allergy School of Specialization in Respiratory Diseases, Federico II University of Naples Naples Italy; ^192^ Centre for Immunology and Infection Control, School of Biomedical Sciences, Faculty of Health Queensland University of Technology Brisbane Australia; ^193^ Office of Research, Metro North Hospital and Health Service Brisbane Australia; ^194^ Department of Medical and Surgical Science University of Foggia Foggia Italy; ^195^ Allergy and Clinical Immunology Centro Regional, Hospital Universitario, Universidad Autónoma de Nuevo Leon Monterrey Mexico; ^196^ Department of Otorhinolaryngology Head and Neck Surgery, University of Crete, School of Medicine Heraklion, Crete Greece; ^197^ Department of Internal Diseases Allergology and Clinical Immunology, Medical University of Silesia in Katowice Katowice Poland; ^198^ Pediatrics Department Universidad Austral de Chile Valvidia Chile; ^199^ School of Health Sciences Catholic University of Salta Salta Argentina; ^200^ Center of Allergy and Immunology, and Georgian Academy of Allergy, Asthma, and Clinical Immunology Tbilisi Georgia; ^201^ Department of Allergy and Clinical Immunology Former President of the Hellenic Society of Allergology and Clinical Immunology, Air Force General Hospital Athens Greece; ^202^ Immunology and Allergy Division, Clinical Hospital University of Chile Santiago Chile; ^203^ Quality Use of Research Medicines Group, Woolcock Institute of Medical Research Macquarie Park New South Wales Australia; ^204^ Macquarie University Macquarie Park New South Wales Australia; ^205^ Faculty of Health, University of Plymouth Plymouth UK; ^206^ Department of Primary Care and Population Health University of Nicosia Medical School Nicosia Cyprus; ^207^ Otolaryngology Department General Hospital of Kalamata Kalamata Greece; ^208^ Division of Clinical Immunology and Allergy, Department of Medicine McMaster University Hamilton Ontario Canada; ^209^ Occupational and Environmental Medicine, Department of Medical Sciences Uppsala University Uppsala Sweden; ^210^ ORL SOKLIC KOSAK Ljubljana Slovenia; ^211^ University Clinic of Respiratory and Allergic Diseases Golnik Slovenia; ^212^ Medical Faculty University of Ljubljana Ljubljana Slovenia; ^213^ Department of Chest Diseases Bagcilar Research and Training Hospital Istanbul Turkey; ^214^ Clinical Management Group Woolcock Institute of Medical Research, Macquarie University Sydney New South Wales Australia; ^215^ Sydney Pharmacy School, Faculty of Medicine and Health University of Sydney Sydney New South Wales Australia; ^216^ Departmental Unit of Allergology Clinical Immunology & Pneumology, Istituto Ospedaliero Fondazione Poliambulanza Brescia Italy; ^217^ Department of Public Health University of Liège Liège Belgium; ^218^ Department of Pneumology GIGA I3 Research Group, University of Liège Liège Belgium; ^219^ Department of Otolaryngology‐Head and Neck Surgery, and Department of Allergy The Third Affiliated Hospital of Sun Yat‐sen University Guangzhou China; ^220^ Allergy Unit, Department of Internal Medicine University Hospital AOU delle Marche Ancona Italy; ^221^ Department of Clinical and Molecular Sciences Marche Polytechnic University Ancona Italy; ^222^ Department of Otorhinolaryngology Head and Neck Surgery Ankara University Medical School Ankara Turkey; ^223^ Department of Otorhinolaryngology Head and Neck Surgery Salzburg Paracelsus Medical University Salzburg Austria; ^224^ Department of Otolaryngology Head and Neck Surgery, Cornell University, Weill Cornell Medical College New York New York USA; ^225^ Allergy & Clinical Immunology Department University Hospital Center “Mother Teresa” Tirana Albania; ^226^ Pneumology Department UMF‐University of Medicine and Pharmacy ‘Carol Davila’, National Institute of Pneumology ‘Marius Nasta’ Bucharest Romania; ^227^ Department of Allergology and Internal Medicine Medical University of Bialystok Bialystok Poland; ^228^ Department of Otolaryngology‐Head and Neck Surgery Johns Hopkins University Baltimore Maryland USA; ^229^ Department of Infection and Immunity Luxembourg Institute of Health Esch‐sur‐Alzette Luxembourg; ^230^ Department of Dermatology and Allergy University Medical Center UZ Brussels, Vrije Universiteit Brussel (VUB) Jette/Brussels Belgium; ^231^ Department of Pharmacotherapy and Pharmaceutical Care Faculty of Pharmacy, Medical University of Warsaw Warsaw Poland; ^232^ Otorhinolaryngology and Neck Surgery Unit San Juan de Dios National Hospital San Miguel El Salvador; ^233^ Allergist, Private Practice Mexico City Mexico; ^234^ Ecole Polytechnique de Palaiseau Palaiseau France; ^235^ IRBA (Institut de Recherche Bio‐Médicale des Armées) Bretigny sur Orge France; ^236^ Université Paris Cité Paris France; ^237^ Center for Allergy and Clinical Immunology Alésia, Paris France; ^238^ Department of Otorhinolaryngology Cliniques Universitaires Saint‐Luc Brussels Belgium; ^239^ Department of Otolaryngology‐Head and Neck Surgery Eye and Ear University Hospital Beirut Lebanon; ^240^ Department of Otorhinolaryngology‐Head and Neck Surgery Dar Al Shifa Hospital Salmiya Kuwait; ^241^ Usher Institute, University of Edinburgh Edinburgh UK; ^242^ Department of Otorhinolaryngology Yamanashi University Yamanashi Japan; ^243^ Mohammed Bin Rashid University (MBRU), Dubai Health Dubai UAE; ^244^ Department of Otorhinolaryngology and Head & Neck Surgery CHU Rangueil‐Larrey Toulouse France; ^245^ Allergy Service University Hospital Professor Polydoro Ernani de São Thiago (HU‐UFSC/EBSERH) Florianopolis Brazil; ^246^ Department of Internal Medicine Federal University of Santa Catarina (UFSC) Florianopolis Brazil; ^247^ Otolaryngology‐HNS, University of Zurich, University Hospital of Zurich Zurich Switzerland; ^248^ Clinic of Allergology and Immunology University Clinical Center of Serbia Belgrade Serbia; ^249^ Faculty of Medicine University of Belgrade Belgrade Serbia; ^250^ Department of Respiratory Medicine Medical Professorial Unit, Tallaght University Hospital & Trinity College Dublin Ireland; ^251^ The Heart and Lung Center Helsinki University Hospital and University of Helsinki Helsinki Finland; ^252^ Department of Immunology and Allergology University Hospital and Faculty of Medicine in Pilsen Plzeň Czech Republic; ^253^ Department of Medicine Solna, Division of Immunology and Respiratory Medicine Karolinska Institutet Stockholm Sweden; ^254^ Department of Clinical Immunology and Transfusion Medicine Karolinska University Hospital Stockholm Sweden; ^255^ Center for Molecular Medicine, Karolinska University Hospital Stockholm Sweden; ^256^ Department of Otorhinolaryngology Heinrich Heine University Düsseldorf, Medical Faculty and University Hospital Düsseldorf Düsseldorf Germany; ^257^ Department of Medicine and Therapeutics The Chinese University of Hong Kong Shatin, N. T. Hong Kong, China; ^258^ ENT and Allergology Düsseldorf Germany; ^259^ Shanghai Skin Disease Hospital, Tongji University School of Medicine Shanghai China

**Keywords:** allergic rhinitis, guidelines, leukotriene receptor antagonists, ocular antihistamines, oral antihistamines

## Abstract

**Background:**

Oral and ocular medications are frequently used in the treatment of allergic rhinitis (AR). As part of the update of the Allergic Rhinitis and its Impact on Asthma (ARIA)‐EAACI guidelines, this manuscript presents the ARIA‐EAACI 2024–2025 recommendations for oral and ocular treatments.

**Methods:**

The ARIA‐EAACI 2024–2025 guideline panel issued recommendations following the Grading of Recommendations, Assessment, Development and Evaluation (GRADE) evidence‐to‐decision framework. Several sources of evidence were used to inform panel judgements and recommendations, including systematic reviews, mHealth and pharmacovigilance data as well as a survey on costs.

**Results:**

Eight guideline questions concerning oral treatments for AR and three questions concerning ocular treatments were addressed. These questions led to the recommendations. Overall, these questions concern the choice between different classes of medication. They also discuss the role of oral antihistamines (OAH), leukotriene receptor antagonists (LTRA), ocular antihistamines (OcAH) and ocular mast cell stabilisers. Four questions had not been previously evaluated in ARIA guidelines, while, for the other four, there was a change in the strength or directionality of the recommendations. Overall, these guidelines recommend using intranasal corticosteroids over OAH and using OAH over LTRA. Moreover, they suggest using OAH over OcAH and suggest being against adding LTRA to OAH. Finally, considerations for choosing between different individual OAHs are presented.

**Conclusion:**

This ARIA‐EAACI 2024–2025 article supports patients, their caregivers and healthcare professionals in choosing oral and ocular treatments for AR. Decisions on treatment should consider the clinical variability of the disease, patients' values and the affordability of medications.

## Graphical abstract

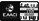

**Abbreviations**
AI: artificial intelligence
AR: allergic rhinitis
ARIA: Allergic Rhinitis and its Impact on Asthma
EAACI: European Academy of Allergy and Clinical Immunology
EtD: evidence‐to‐decision framework
GRADE: Grading of Recommendations, Assessment, Development and Evaluation
INAH: intranasal antihistamine
INCS: intranasal corticosteroid
LTRA: leukotriene receptor antagonist
NMA: network meta‐analysis
OAH: oral antihistamine
OcAH: ocular antihistamine
PAR: perennial allergic rhinitis
RCT: randomised controlled trial
SAR: seasonal allergic rhinitis
WHO: World Health Organisation

## 1 Introduction

Allergic rhinitis (AR) is a highly prevalent and burdensome disease [1, 2, 3, 4, 5]. Over the past two decades, clinical practice guidelines have supported healthcare providers and patients in managing AR effectively. Among these, the Allergic Rhinitis and its Impact on Asthma (ARIA) initiative first published its guidelines in 2001 [6], with updates in 2008 [7], 2010 [8], 2016 [9] and 2020 [10]. These updates reflected the development of new treatments and/or methodological improvements, including the adoption of the Grading of Recommendations, Assessment, Development and Evaluation (GRADE) approach in 2010 and 2016 [9], and the inclusion of MASK‐air mHealth data in 2020 [10].

The new revision of the ARIA guidelines (ARIA‐EAACI 2024–2025) has been endorsed by the European Academy of Allergy and Clinical Immunology (EAACI). It aims to respond to emerging evidence and evolving clinical needs. In recent years, evidence from other data sources, particularly mHealth, has expanded our understanding of AR beyond traditional randomised controlled trials (RCTs) [11], enabling a more comprehensive evaluation of the acceptability of AR treatments, cost‐effectiveness of the interventions and patients' values associated with AR. In particular, this has been made possible as mHealth evidence has shed light on medication use patterns and adherence [12, 13], patients' satisfaction with treatments [14], utilities associated with each treatment (Lourenço‐Silva et al., under review) and the impact of AR on work productivity [3]. A comprehensive description on how mHealth data have been used to support these guidelines can be found elsewhere [15, 16].

Considering the above, the ARIA‐EAACI 2024–2025 guidelines have been conceived as person‐centred, digitally enabled and assisted by artificial intelligence (AI), using the GRADE approach [15]. This emphasis on a person‐centred guideline is relevant due to inter‐individual variability in (i) exposure and responses to triggers/allergens, (ii) impact of AR on daily life, (iii) values in relation to rhinitis health states and (iv) disease management. The use of AI has supported the identification of patient‐centred guideline questions [17] and of potentially relevant outcomes (for a detailed discussion see [15, 16]).

The first set of recommendations from the ARIA‐EAACI 2024–2025 guidelines focused on intranasal treatments for AR [18].

This report presents the recommendations on oral and ocular treatments of AR. Most recommendations concern oral H_1_‐antihistamines (OAH), leukotriene receptor antagonists (LTRA) and ocular H_1_‐antihistamines (OcAH). The target audience of these guidelines includes, among others, patients with AR, health professionals managing adults or children with AR, social workers and health policymakers.

## 2 Questions Addressed by This Guideline

In ARIA‐EAACI 2024–2025, 42 questions on AR management were voted by guideline panel members as “prioritised questions” [17]. Among these, nine concerned oral and ocular AR treatments and are addressed by this report. Two additional questions are addressed in this guideline as the panel judged them assufficiently important to be included: (i) should OAH vs. LTRA be used for the treatment of AR? (added because there were questions comparing OAH vs. no treatment, and LTRA vs. no treatment); and (ii) should OcAH vs. ocular mast cell stabilisers be used for the treatment of ocular symptoms in patients with AR? (added because mast cell stabilisers are widely used in low‐ and middle‐income countries). The full list of questions is presented in Table 1, alongside their corresponding recommendations and capsule justifications.

**TABLE 1:** Recommendations of the ARIA‐EAACI 2024–2025 guidelines for the prioritised questions on oral and ocular treatments.

Recommendation	Capsule justification	Subgroup considerations	Implementation considerations
(A) New questions in ARIA 2024–2025			
Should any specific OAH vs. other OAH be used for the treatment of AR?			
In patients with AR, we suggest that the choice of specific OAH over others should be based on patients' preferences on efficacy and safety and on availability and affordability. (Conditional recommendation|Very Low CoE)	Bilastine, ebastine, rupatadine and cetirizine are effective OAHs, with rupatadine acting rapidly and cetirizine being affordable yet sedating. Loratadine and fexofenadine are non‐sedating and in the WHO List of Essential Medicines, albeit the latter is less effective.	In preschool‐ and school‐aged children, evidence was not sufficient to support a specific recommendation.	Cost and availability may influence treatment choices. Bilastine and fexofenadine may be preferred in the case of renal/hepatic impairment. Locally produced medications may help reduce the environmental impact.
Should an OAH + LTRA vs. OAH be used for the treatment of AR?			
In patients with AR, we suggest using OAH over OAH + LTRA. (Conditional recommendation|Moderate CoE)	OAH + LTRA show no added clinically relevant benefit over OAH alone and result in increased costs. In addition, there are important safety concerns with LTRA (montelukast).	Recommendation applicable to preschool‐ and school‐aged children.	In LMIC, OAH in the WHO List of Essential Medicines or locally produced generics may be considered.
Should OcAH vs. OAH be used for the treatment of ocular symptoms in patients with AR?			
In patients with AR, we suggest **against** using OcAH over OAH, except for very fast relief of ocular symptoms (Conditional recommendation|Very Low CoE)	Compared to OcAH, OAH are more efficacious and associated with higher acceptability. In addition, OAH tend to be more affordable with some being in the WHO List of Essential Medicines.	Recommendation applicable to (i) preschool‐ and school‐aged children, and (ii) patients with allergic conjunctivitis without nasal symptoms.	In LMIC, OAH in the WHO List of Essential Medicines or locally produced generics may be considered.
Should OcAH vs. ocular mast cell stabilisers be used for the treatment of ocular symptoms in patients with AR?			
In patients with AR, we suggest using OcAH over mast cell stabilisers (Conditional recommendation|Very Low CoE)	OcAH and ocular mast cell stabilisers are similar in terms of efficacy and safety. However, OcAH are associated with a faster onset of action and fewer applications, and are probably cost‐effective in a wide set of countries.	Recommendation applicable to preschool‐ and school‐aged children.	Mast cell stabilisers may be considered in countries where ocular antihistamines are not available or are less affordable.
(B) Questions with changed recommendation in terms of strength or directionality (compared to ARIA 2010/2016)			
Should OAH versus LTRA be used for the treatment of AR?			
In patients with AR, we recommend using OAH over LTRA. (Strong recommendation|Moderate CoE)	OAH offer only a trivial added benefit over LTRA in symptom relief, but have a more favourable safety profile. LTRA have important safety concerns, and OAH are generally more accessible.	Recommendation applicable to preschool‐ and school‐aged children.	In LMIC, OAH in the WHO List of Essential Medicines or locally produced generics may be considered.
Should INCS versus OAH be used for the treatment of AR?			
In patients with AR, we recommend using INCS over OAH. (Strong recommendation|Moderate CoE)	INCS are more effective in improving nasal symptoms and quality of life. INCS and OAH display a similar safety profile. INCS tend to be cost‐effective and are associated with higher satisfaction.	Recommendation applicable to preschool‐ and school‐aged children. In pregnant women, triamcinolone may have teratogenic effects.	In LMIC, INCS in the WHO List of Essential Medicines or locally produced generics may be considered.
Should LTRA vs. no treatment be used for the treatment of AR?			
In patients with AR under no treatment, we suggest **against** using LTRA. (Conditional recommendation|Moderate CoE).	LTRA offer small improvements in seasonal AR, with limited evidence in perennial AR. LTRA (montelukast) have rare but important neuropsychiatric safety concerns and there are safer alternatives for patients under no treatment.	Recommendation applicable to preschool‐ and school‐aged children.	LTRA may be considered in patients who are not well‐controlled with other medications and have a strong preference for oral treatments (particularly if they have asthma).
Should OcAH versus no treatment be used for the treatment of ocular symptoms in patients with AR?			
In patients with seasonal AR, we suggest **against** using OcAH over no treatment, except for few days (7 or less) or as‐needed for very fast symptom relief (Conditional recommendation|Low CoE). In patients with perennial AR, we suggest using OcAH over no treatment (Conditional recommendation|Very Low CoE).	In perennial AR, the desirable effects outweigh the risks for undesirable effects but the same does not happen in seasonal AR. In addition, OcAH are associated with moderate costs and with impacts on equity and planetary health.	Recommendation applicable to preschool‐ and school‐aged children.	This question is not focused on adding OcAH to a previous treatment
(C) Other questions in ARIA 2024–2025			
Should OAH versus no treatment be used for the treatment of AR?			
In patients with AR, we recommend using OAH over no treatment. (Strong recommendation|Moderate CoE)	OAH are effective in improving nasal symptoms, ocular symptoms and quality‐of‐life. OAH are overall safe, cost‐effective and well accepted by patients.	Recommendation applicable to preschool‐ and school‐aged children.	In LMIC, OAH in the WHO List of Essential Medicines or locally produced generics may be considered.
Should second‐generation OAH versus first‐generation OAH be used for the treatment of AR?			
In patients with AR, we recommend using second‐generation OAH over first‐generation OAH. (Strong recommendation|Very low CoE).	Evidence comparing first‐ and second‐generation OAH is limited. Second‐generation agents are safer (in particular, resulting in less sedation) and associated with better adherence and higher patient satisfaction.	Recommendation applicable to preschool‐ and school‐aged children.	In LMIC, second‐generation OAH in the WHO List of Essential Medicines or locally produced generics may be considered.
Should INAH versus OAHs be used for the treatment of AR?			
In patients with AR, we suggest either using INAH or OAH (Conditional recommendation|Moderate CoE)	INAH are more effective in improving nasal symptoms and quality of life. However, OAH are associated with a lower risk of adverse events, are more widely available and cost‐effective. OAH are associated with higher treatment satisfaction and better adherence.	Recommendation applicable to preschool‐ and school‐aged children.	None specific

Abbreviations: AR, allergic rhinitis; CoE, certainty of evidence; INAH, intranasal antihistamines; INCS, intranasal corticosteroids; LMIC, low‐ and middle‐income countries; LTRA, leukotriene receptor antagonists; OAH, oral H_1_‐antihistamines; OcAH, ocular H_1_‐antihistamines.

## 3 Methodology

A detailed description of the methods used to develop these recommendations is available elsewhere [16, 18], with a brief overview being provided in the online supplement.

### 3.1 How to Use These Guidelines

The ARIA guidelines are not intended to impose a mandate or standard of care for individual countries, but rather to provide a basis for rational, informed decisions. Recommendations provide guidance for typical patients but cannot account for all unique individual circumstances. Thus, clinicians are encouraged to individualise their practice ‐ considering the clinical presentation of each patient and the specificities of the local context ‐ and to reach decisions via shared decision‐making.

For each question, in accordance with GRADE, we issued either a “strong” or “conditional” recommendation (terminology clarification in Box 1). The wording of the recommendations reflects their strength, with “we recommend” implying a strong recommendation and “we suggest” implying a conditional recommendation. In each recommendation, we present information on the certainty of evidence across the different outcomes of interest (quality of the whole body of evidence, considering altogether desirable and undesirable effects; Box 1). Finally, to support panel members in formulating guideline recommendations, effect sizes were categorised as “trivial or none”, “small”, “moderate” or “large”. This terminology will be used throughout this report and is clarified in Box 1.

BOX 1 **Clarification of the terminology used in these guidelines.**

**Table:** 

*Strength of recommendations*: Strong recommendation ○ *For patients*: Most patients in this situation would want the recommended course of action, and only a small proportion would not. ○ *For clinicians*: Most patients should receive the intervention. Adherence to a strong recommendation could be used as a quality criterion or performance indicator. Formal decision aids are not likely to be needed to help patients make decisions consistent with their values and preferences. ○ *For healthcare policy makers*: The recommendation can be adopted as a policy or performance measure in most situations Conditional recommendation ○ *For patients*: Most patients in this situation would want the suggested course of action, but many would not. ○ *For clinicians*: Recognise that different choices will be appropriate for individual patients and that you must help each patient arrive at a management decision consistent with his or her values and preferences. Decision aids might be useful in helping patients to make decisions consistent with their values and preferences. ○ *For healthcare policy makers*: Policy making will require substantial debate and involvement of various stakeholders. Documentation of appropriate (e.g., shared) decision‐making processes can serve as a performance measure. *Certainty of evidence*: The certainty of evidence concerns how certain we are that the observed magnitude of desirable and undesirable anticipated effects lies on one side of a specified threshold or within a chosen range (reflecting the “quality” of available evidence). The certainty of evidence can be classified as “very low”, “low”, “moderate” or “high”. The certainty of evidence is independent of the directionality of the recommendation and of the effect sizes of the associations. *Categorisation of the effect sizes*: The magnitude of the anticipated desirable and undesirable effects (“benefits and harms”) is classified by the GRADE working group as “trivial or none”, “small [but meaningful]”, “moderate” or “large”. A trivial effect is observed when the magnitude of the effects is so small that it is not sufficiently important in terms of anticipated health consequences. Non‐trivial effects can be considered “small [but meaningful]”, “moderate” or “large” depending on the magnitude of effect sizes. Of note, for continuous outcomes, effects were classified as “trivial or none”, “small”, “moderate” or “large” based on their meta‐analytical standardised mean differences (the cutoff values of 0.2, 0.5 and 0.8 were used for decision thresholds). For dichotomous outcomes, we used as decision thresholds the values of 65, 161 and 303 events per 1000 participants (defining respectively what corresponds to a small, moderate and large effect) [19].

In this manuscript, we provide only a summary of the evidence underlying each recommendation (“brief justification”). The full Evidence‐to‐Decision (EtD) frameworks for each question can be found online (links provided below alongside each question).

## 4 Recommendations and Summary of Findings

Table 1 outlines the recommendations for each addressed question. In the subsequent sections, we provide the rationale for each recommendation. Of note, we do not discuss patients' values and preferences individually for each question, as these were consistent across all topics. Specifically, patients with AR generally (i) place greater importance on the efficacy of AR interventions than on their safety and (ii) consider nasal symptoms (in particular, nasal congestion) to have the greatest impact [20]. Also, please note that whenever mentioning OAH, unless otherwise specified (i.e., in recommendation 10), we are referring to “second‐generation OAH”. Table 2 presents, for each question, the judgement of the effect size and the certainty of the evidence for each outcome.

**TABLE 2:** Judgements on the effect sizes and certainty of evidence (CoE) assessments for each outcome in each prioritised question comparing each intervention to a comparator.

Question		Seasonal allergic rhinitis					Perennial allergic rhinitis				
		Nasal symptoms	Ocular symptoms	Quality of life	AE	Serious AE	Nasal symptoms	Ocular symptoms	Quality of life	AE	Serious AE
Should any specific OAH versus other OAH be used for the treatment of AR?	Effect size	Small	Small	Small	Trivial	—^b^	Small	—^b^	Small	Trivial	—^b^
	CoE	Very low/low^a^	Very low/low^a^	Very low^a^	Very low/low^a^	—^b^	Low/moderate^a^	—^b^	Moderate	Very low/low^a^	—^b^
Should OAH + LTRA versus OAH be used for the treatment of AR?	Effect size	Trivial	Trivial	Trivial	Trivial	Trivial	—^b^	—^b^	—^b^	—^b^	—^b^
	CoE	High	Low	High	Moderate	Moderate	—^b^	—^b^	—^b^	—^b^	—^b^
Should OcAH versus OAH be used for the treatment of ocular symptoms in AR?	Effect size	—^b^	Small	—^b^	Small	—^b^	—^b^	—^b^	—^b^	Small	—^b^
	CoE	—^b^	Very low	—^b^	Very low	—^b^	—^b^	—^b^	—^b^	Very low	—^b^
Should OcAH versus MCS be used for the treatment of ocular symptoms in AR?	Effect size	—^b^	Trivial	—^b^	Trivial	—^b^	—^b^	—^b^	—^b^	—^b^	—^b^
	CoE	—^b^	Very low	—^b^	Very low	—^b^	—^b^	—^b^	—^b^	—^b^	—^b^
Should OAH versus LTRA be used for the treatment of AR?	Effect size	Trivial	Trivial	Trivial	Trivial	Trivial	Trivial	—^b^	Trivial	—^b^	—^b^
	CoE	High	Low	High	Moderate	Moderate	Moderate	—^b^	Very low	—^b^	—^b^
Should INCS versus OAH be used for the treatment of AR?	Effect size	Small	Trivial	Moderate	Trivial	—^b^	Small	—^b^	Small	Small	—^b^
	CoE	Moderate	Moderate	Very low	High	—^b^	Moderate	—^b^	Very low	High	—^b^
Should LTRA versus no treatment be used for the treatment of AR?	Effect size	Small	Small	Small	Trivial	Trivial	Trivial	—^b^	Small	—^b^	—^b^
	CoE	Moderate	Low	Moderate	Moderate	Moderate	Moderate	—^b^	Very low	—^b^	—^b^
Should OcAH versus no treatment be used for the treatment of ocular symptoms in AR?	Effect size	—^b^	Trivial	—^b^	Trivial	—^b^	—^b^	Large	—^b^	Small	—^b^
	CoE	—^b^	Low	—^b^	Low	—^b^	—^b^	Very low	—^b^	Very low	—^b^
Should OAH versus no treatment be used for the treatment of AR?	Effect size	Small	Small	Small	Trivial	Trivial	Small	—^b^	Small	Trivial	Trivial
	CoE	High	Moderate	High	Moderate	Moderate	High	—^b^	Moderate	Moderate	Moderate
Should second‐generation OAH versus first‐generation OAH be used for the treatment of AR?	Effect size	Small	—^b^	Trivial	Small	—^b^	—^b^	—^b^	—^b^	—^b^	—^b^
	CoE	Very low	—^b^	Low	Very low	—^b^	—^b^	—^b^	—^b^	—^b^	—^b^
Should INAH versus OAH be used for the treatment of AR?	Effect size	Small	Trivial	Small	Small	—^b^	Trivial	—^b^	Small	Small	—^b^
	CoE	Moderate	Moderate	Moderate	Low	—^b^	Moderate	—^b^	Moderate	Low	—^b^

Abbreviations: AE, adverse events; AR, allergic rhinitis; INAH, intranasal antihistamines; INCS, intranasal corticosteroids; LTRA, leukotriene receptor antagonists; MCS, mast cell stabilisers; OAH, oral H_1_‐antihistamines; OcAH, Ocular H1‐antihistamines. Colour code: Green=High certainty of evidence; Yellow=Moderate certainty of evidence; Orange=Low certainty of evidence; Red=Very low certainty of evidence; Blue=Effect size (the darker the shade of blue, the larger the effect size).

^a^ Most common CoE assessments for the considered comparisons.

^b^ No available evidence.

**A. New questions in ARIA‐EAACI 2024–2025**

**1. Should any specific individual second‐generation oral** **H**_**1**_ **‐antihistamines vs. other individual oral** **H**_**1**_ **‐antihistamines be used for the treatment of allergic rhinitis?**

**Link for the full EtD:** https://aria.med.up.pt/etd_oral‐01/

**Context:** There are several second‐generation OAH available, rendering it important to provide recommendations not only at a class level but also in relation to individual OAH.

**Recommendation: In patients with AR, we suggest that the choice of specific second‐generation OAHs over others should be based on patients' preferences on efficacy and safety and on availability and affordability (conditional recommendation based on mostly very low certainty of evidence)**.

- Considerations in children, adolescents and pregnant women: In preschool‐ and school‐age children, evidence was not sufficient to support recommending a specific second‐generation OAH. In pregnant women, existing studies do not suggest a teratogenic effect of OAH and the Food and Drug Administration (FDA) considers loratadine and cetirizine generally safe in pregnancy [21].
- Implementation considerations: When selecting a second‐generation OAH, several factors should be considered, including efficacy, safety (e.g., sedative potential), affordability and specific patient characteristics. Patients often value efficacy over safety; however, the sedative effects of some OAH can influence adherence and daily functioning, making the balance between efficacy and safety clinically relevant. Moreover, in some professions, workers cannot have medications with sedative effect. Table 3 displays a summary of implementation and safety aspects. Some highlights include:
  ○ Cetirizine consistently ranks high in terms of efficacy and patient satisfaction. It is affordable in most countries and included in the World Health Organisation (WHO) List of Essential Medicines. Nonetheless, it has a relatively higher sedative potential compared to other second‐generation OAH and may be better suited for evening use. In addition, the FDA has warned that cetirizine (and levocetirizine) can rarely result in severe itching after discontinuation.
  ○ Loratadine offers good efficacy, is non‐sedating, widely affordable, and also included in the WHO List of Essential Medicines.
  ○ Fexofenadine, also in the WHO List of Essential Medicines, is considered non‐sedating but appears to be less efficacious than other OAH.
  ○ Bilastine, ebastine and rupatadine are among the most efficacious second‐generation OAHs and are generally well‐accepted by patients. Rupatadine has a rapid onset of action. However, the affordability of these medications can vary depending on the setting, potentially limiting their use in low‐ and middle‐income countries.
  ○ In terms of renal or hepatic impairment, bilastine and fexofenadine are preferred due to the absence of required dose adjustments. However, fexofenadine and bilastine blood levels (as well as those of rupatadine) may be reduced because of interactions with grapefruit and some other fruit juices [22], so they should be taken with water (Table 3) [23].
  ○ In low‐ and middle‐income countries, the choice of OAH is often shaped by local availability and cost.
  ○ Planetary health concerns may also play a role, encouraging the selection of locally manufactured generics to minimise environmental impact.

**TABLE 3:** Considerations of affordability, adjustments and interactions of second‐generation oral antihistamines.

	WHO list of essential medicines	Sedative effect	Need for adjustments		Interactions		Electrocardiographic changes described as rare AEs
			Renal impairment	Liver impairment	Fruit juices	Other medications	
Bilastine	No	No	No	No	Yes	Yes	Yes
Cetirizine	Yes	Moderate	Yes	If renal impairment	No	No	No
Desloratadine	No	Low	No	No	No	No	Yes
Ebastine	No	No	No	No	No	Yes	No
Fexofenadine	Yes	No	No	No	Yes	Yes	No
Levocetirizine	No	Moderate	Yes	If renal impairment	No	No	No
Loratadine	Yes	Low	No	Yes	No	Yes	No
Rupatadine	No	Low	Possibly	Possibly	Yes	Yes	No

Abbreviations: AE, adverse events; WHO, World Health Organisation. Colour code: Green=Desirable; Yellow=Partly undesirable; Red=Undesirable.

**Brief justification:**

- Efficacy and safety:
  ○ A network meta‐analysis (NMA) [24] suggested that, in seasonal AR (SAR), cetirizine, desloratadine, ebastine, loratadine, olopatadine and rupatadine were associated with a high probability of clinically meaningful improvement in nasal symptoms. Among these, cetirizine, ebastine and rupatadine had the highest probability of being the most effective. In perennial AR (PAR), all OAHs except loratadine were superior to placebo, with rupatadine showing the highest probability of being the most effective, followed by ebastine.
  ○ In SAR, desloratadine, rupatadine, bilastine, loratadine and cetirizine were among the OAHs with the highest probability of improving ocular symptoms. All OAHs except fexofenadine showed a clinically meaningful improvement compared to placebo. Loratadine had the highest probability of being the most effective. In PAR, no studies were available on ocular symptoms.
  ○ In terms of rhinoconjunctivitis‐related quality of life (RQLQ), in SAR, desloratadine, fexofenadine, loratadine, olopatadine and rupatadine were the OAHs with the highest probability of improving quality of life. All OAHs showed significant improvement compared to placebo. Among medications assessed in more than one study, desloratadine had the highest probability of achieving a moderate improvement. In PAR, cetirizine, desloratadine, levocetirizine and rupatadine were all more effective than placebo, with levocetirizine and cetirizine showing the highest probability of a clinically meaningful benefit. Levocetirizine and desloratadine were the OAHs most likely to be the most effective in improving quality of life.
  ○ Similar frequencies and patterns of adverse events and serious adverse events were observed with the different second‐generation OAHs based on data from RCTs and pharmacovigilance.
- Resources required, cost‐effectiveness and equity: A survey of ARIA experts reported that the least and most expensive OAH vary widely across countries, but that loratadine, cetirizine and levocetirizine are most frequently the least expensive OAH. We did not identify any cost‐effectiveness study comparing OAH. However, the cost data from our survey, combined with EQ‐5D data from MASK‐air indicate that bilastine, fexofenadine, loratadine, rupatadine and desloratadine are the OAH most frequently found to be cost‐effective compared to others. Cetirizine, fexofenadine and loratadine are in the WHO List of Essential Medicines.
- Acceptability and feasibility: Observational studies suggest that levocetirizine and cetirizine are associated with the highest levels of patient and physician satisfaction among OAH. Fexofenadine also performed well, particularly in paediatric populations, with high satisfaction scores from both parents and physicians. MASK‐air data suggest that OAH are generally associated with similar levels of treatment satisfaction. However, desloratadine was associated with slightly higher satisfaction than bilastine, levocetirizine and loratadine. Fexofenadine and ebastine were more often used in co‐medication than cetirizine, desloratadine and loratadine, suggesting possible differences in perceived effectiveness in monotherapy.
- Planetary health: No specific evidence was found in terms of comparative impact on planetary health.

**2. Should a combination of a leukotriene receptor antagonist and an oral H_1_ ‐antihistamine vs. an oral H_1_ ‐antihistamine alone be used for the treatment of allergic rhinitis?**

**Link for the full EtD:** https://aria.med.up.pt/etd_oral‐02/

**Context:** Patients taking OAH often resort to co‐medication with LTRA, prompting a need for assessing the added value of such co‐medication strategy.

**Recommendation: In patients with AR, we suggest using OAH alone over combinations of OAH and LTRA (conditional recommendation based on moderate certainty of evidence)**.

- Considerations in children and adolescents: The recommendation is applicable to preschool and school‐aged children
- Implementation considerations: In low‐ and middle‐income countries (LMIC), OAH in the WHO List of Essential Medicines and/or locally produced generics may be preferred.

**Brief justification:**

- Efficacy and safety:
  ○ A NMA (data in the EtD link) found that, in SAR, the combined use of OAH and LTRA does not improve nasal or ocular symptoms or RQLQ compared with OAH alone. In PAR, no studies were available.
  ○ Regarding safety, a NMA of RCTs did not find differences between OAH + LTRA versus OAH alone. Serious adverse events were rare and not treatment‐related. However, observational studies and pharmacovigilance data have raised concerns about rare neuropsychiatric adverse events associated with LTRA, such as insomnia, mood changes and suicidal ideation, leading to an FDA black box warning [25].
- Resources required, cost‐effectiveness and equity: OAH + LTRA are more expensive than OAH alone. We did not identify any cost‐effectiveness study comparing these two interventions. Some OAH are in the WHO List of Essential Medicines (cetirizine, fexofenadine and loratadine), contrary to LTRA.
- Acceptability and feasibility: MASK‐air data suggest that OAH + LTRA are associated with slightly higher adherence and greater treatment satisfaction compared to OAH alone.
- Planetary health: No specific evidence was found in terms of comparative impact on planetary health. However, the use of OAH + LTRA, compared to OAH alone, implies the use of additional resources with environmental impact.

**3. Should ocular H_1_ ‐antihistamines vs. oral H_1_ ‐antihistamines be used for the treatment of ocular symptoms in patients with allergic rhinitis?**

**Link for the full EtD:** https://aria.med.up.pt/etd_ocular‐01/

**Context:** OAH are frequently used not only to control nasal but also ocular symptoms. However, some patients may prefer OcAH due to their faster onset of action.

**Recommendation: In patients with AR, we suggest against using OcAH over OAH except for very fast relief of ocular symptoms (conditional recommendation based on very low certainty of evidence)**.

- Considerations in children and adolescents: The recommendation is applicable to preschool and school‐aged children.
- Implementation considerations: In LMIC, OAH in the WHO List of Essential Medicines and/or locally produced generics may be preferred.

**Brief justification:**

- Efficacy and safety:
  ○ A NMA based on previously conducted systematic reviews [26, 27] (data on the EtD link) found that, compared with OcAH, OAH are associated with a small but important improvement in ocular symptoms in patients with SAR. In PAR, no studies were available.
  ○ Regarding safety, a NMA of RCTs found that OcAH are associated with a higher risk of adverse events than OAH both in SAR and PAR (small but important effect). Serious adverse events were rare and not treatment‐related.
- Resources required, cost‐effectiveness and equity: A survey of ARIA experts reported OcAH to be more expensive than OAH in 39 out of 44 countries for which data were available. Considering the results of effectiveness studies, it is likely that OcAH are not cost‐effective. Three OAH—cetirizine, fexofenadine and loratadine—are in the WHO List of Essential Medicines, but this is not the case with any OcAH. In addition, OAH are available in more countries than OcAH.
- Acceptability and feasibility: MASK‐air data suggest that OAH are associated with higher adherence and lower odds of being used as co‐medication (a proxy for poor rhinitis control). OAH and OcAH are associated with similar treatment satisfaction. However, OcAH displays a faster onset of action.
- Planetary health: No specific evidence comparing OAH and OcAH in terms of impact on planetary health was found. OcAH frequently come in small plastic vials with short expiry time windows—their occasional use may result in relevant waste.

**4. Should ocular H_1_ ‐antihistamines vs. ocular mast cell stabilisers be used for the treatment of ocular symptoms in patients with allergic rhinitis?**

**Link for the full EtD:** https://aria.med.up.pt/etd_ocular‐02/

**Context:** Ocular antihistamines and mast cell stabilisers (chromones) are two medication classes which are frequently topically used to control ocular symptoms.

**Recommendation: In patients with AR, we suggest using OcAH over ocular mast cell stabilisers (conditional recommendation based on very low certainty of evidence)**.

- Considerations in children and adolescents: The recommendation is applicable to preschool and school‐aged children.
- Implementation considerations: None

**Brief justification:**

- Efficacy and safety:
  ○ In SAR, identified studies suggested trivial differences when comparing the improvement of ocular symptoms between OcAH and ocular mast cell stabilisers. In PAR, no studies were available.
  ○ The identified trials in SAR suggested trivial differences in the frequency of adverse events when comparing OcAH and ocular mast cell stabilisers. Serious adverse events were not reported in the identified trials. In PAR, no studies were available.
- Resources required, cost‐effectiveness and equity: A survey of ARIA experts suggested OcAH to be more expensive than ocular mast cell stabilisers in 20 out of 34 countries for which data were available. In most countries where OcAH are more expensive, they were found to be cost‐effective in relation to ocular mast cell stabilisers. No OcAH or ocular mast cell stabiliser is in the WHO List of Essential Medicines.
- Acceptability and feasibility: MASK‐air data suggest that OcAH are associated with higher odds of being used as co‐medication. However, compared to mast cell stabilisers, OcAH have a faster onset of action and require fewer uses per day (which can make them more acceptable to patients).
- Planetary health: No specific evidence was found in terms of comparative impact on planetary health.

**B. Questions with a change in recommendation directionality and/or strength in ARIA‐EAACI 2024–2025**.

**5. Should oral H_1_ ‐antihistamines vs. leukotriene receptor antagonists be used for the treatment of allergic rhinitis?**

**Link for the full EtD:** https://aria.med.up.pt/etd_oral‐03/

**Context:** OAH and LTRA may both be used for the treatment of AR. While OAH are widely used as first‐line treatment, LTRA are often used in patients with coexisting asthma or when OAH are not well tolerated.

**Recommendation: In patients with AR, we recommend using OAH over LTRA (strong recommendation based on moderate certainty of evidence)**.

- Considerations in children and adolescents: The recommendation is applicable to preschool and school‐aged children.
- Implementation considerations: In LMIC, OAH in the WHO List of Essential Medicines and/or locally produced generics may be preferred.

**Change in recommendation from previous ARIA guidelines:** Previous ARIA guidelines suggested using either OAH or LTRA (conditional recommendation) in patients with SAR, and suggested using OAH over LTRA (conditional recommendation) in patients with PAR.

**Brief justification:**

- Efficacy and safety:
  ○ A NMA found that OAH were associated with greater improvement in nasal symptoms compared to LTRA in both SAR and PAR (data in the EtD link). However, the differences displayed a high probability of being trivial.
  ○ For ocular symptoms, OAH resulted in trivial improvements compared to LTRA in SAR. No studies were available for PAR.
  ○ Regarding RQLQ, we found trivial differences between OAH and LTRA in either SAR or PAR.
  ○ We found no significant difference in the frequency of adverse events between OAH and LTRA in SAR. No evidence was available for PAR. However, observational studies and pharmacovigilance data have raised concerns about rare neuropsychiatric effects associated with LTRA, including depression, anxiety and suicidal ideation, which led to a black box warning issued by the FDA [25].
- Resources required, cost‐effectiveness and equity: A survey of ARIA experts reported that OAH and LTRA are widely available. In 46 out of the 48 countries with available data, OAH are cheaper than LTRA. Limited utility data prevented cost‐effectiveness analyses. LTRA are not included in the WHO List of Essential Medicines, while three OAHs (cetirizine, fexofenadine and loratadine) are.
- Acceptability and feasibility: MASK‐air data suggest that satisfaction with LTRA may be higher than with OAH, though data for LTRA were sparse. Co‐medication was more frequent with LTRA (83.0%) than with OAH (50.1%).
- Planetary health: No specific evidence was found in terms of comparative impact on planetary health.

**6. Should intranasal corticosteroids vs. oral H_1_ ‐antihistamines be used for the treatment of allergic rhinitis?**

**Link for the full EtD:** https://aria.med.up.pt/etd_oral‐04/

**Context:** Intranasal corticosteroids (INCSs) and OAHs are widely used for the treatment of AR. While INCS have been described as more effective, OAH have some advantages, including their onset of action and route of administration.

**Recommendation: In patients with AR, we recommend using INCS over OAH (strong recommendation based on moderate certainty of evidence)**.

- Considerations in children, adolescents and pregnant women: The recommendation is applicable to preschool‐aged, school‐aged children and pregnant women. However, for pregnant women, there are concerns that one INCS, triamcinolone, may have teratogenic effects.
- Implementation considerations: In LMIC, INCS in the WHO List of Essential Medicines and/or locally produced generics may be preferred.

**Change in recommendation from previous ARIA guidelines:** Previous ARIA guidelines suggested using INCS over OAH (conditional recommendation).

**Brief justification:**

- Efficacy and safety:
  ○ A pairwise meta‐analysis comparing INCS versus OAH on nasal symptoms included 15 RCTs in SAR [28]. INCS were associated with an improvement in nasal symptoms (68% probability of a meaningful difference). Consistent results were observed when INCS and OAH were compared in a NMA (data in the EtD link). For PAR, no RCTs were identified directly comparing INCS versus OAH. Indirect comparisons from a NMA indicated that INCS were associated with improved nasal symptoms compared to OAH (data in the EtD link).
  ○ For ocular symptoms in patients with SAR, a pairwise meta‐analysis of five RCTs revealed that INCS displayed a 21% probability of a larger improvement compared to OAH [28]. Consistent results were observed in a NMA (data in the EtD link). No evidence was obtained for PAR.
  ○ Regarding RQLQ, a pairwise meta‐analysis comparing INCS versus OAH included two RCTs in SAR [28]. INCS were associated with an improvement in RQLQ (100% probability of a meaningful difference). Consistent results were observed when INCS and OAH were compared in a NMA (data in the EtD link). For PAR, no RCTs were identified directly comparing INCS versus OAH. Indirect comparisons from a NMA indicated that INCS were associated with a greater RQLQ improvement compared to OAH, although the difference was smaller than that observed for SAR (data in the EtD link).
  ○ Regarding safety, both a pairwise meta‐analysis [28] and a NMA pointed to a trivial difference between INCS and OAH in terms of adverse events. For PAR, no RCTs were identified directly comparing INCS versus OAH. Indirect comparisons from a NMA indicated that the impact of INCS may range from a small decrease to a moderate increase in the frequency of adverse events compared to OAH (data in the EtD link).
- Resources required, cost‐effectiveness and equity: A survey of ARIA experts reported that INCS and OAH are widely available. In 35 out of the 51 countries with available data, OAH are cheaper than INCS. However, based on utilities computed using MASK‐air data, INCS would be cost‐effective in most countries. One INCS (budesonide) and three OAHs (cetirizine, fexofenadine and loratadine) are included in the WHO List of Essential Medicines.
- Acceptability and feasibility: MASK‐air data suggest that INCS are associated with higher treatment satisfaction compared to OAH [14]. However, INCS are more frequently used in co‐medication than OAH. OAH have a faster onset of action compared to INCS (median of 60 min versus 720 min, respectively).
- Planetary health: No specific evidence was found in terms of comparative impact on planetary health.

**7. Should leukotriene receptor antagonists vs. no treatment be used for the treatment of allergic rhinitis?**

**Link for the full EtD:** https://aria.med.up.pt/etd_oral‐05/

**Context:** LTRA are sometimes used for the treatment of AR, particularly if there is comorbid asthma. However, some safety concerns have emerged.

**Recommendation: In patients with AR under no treatment, we suggest against using LTRA (conditional recommendation based on moderate certainty of evidence)**.

- Considerations in children and adolescents: The recommendation is applicable to children and adolescents.
- Implementation considerations: LTRA may be considered in patients who are not well‐controlled with other medications and have a strong preference for oral treatments (particularly if they have asthma). In patients who are not using any treatment, the ARIA guideline panel suggests against using LTRA as the starting medication.

**Change in recommendation from previous ARIA guidelines:** Previous ARIA guidelines suggested using LTRA over no treatment in SAR (conditional recommendation), but suggested against its use in PAR.

**Brief justification:** LTRA have rare but important neuropsychiatric safety concerns and there are safer alternatives for patients under no treatment. See online supplement for more details.

**8. Should ocular H_1_ ‐antihistamines vs. no treatment be used for the treatment of ocular symptoms in patients with allergic rhinitis?**

**Link for the full EtD:** https://aria.med.up.pt/etd_ocular‐03/ and https://aria.med.up.pt/etd_ocular‐04/

**Context:** OcAH are frequently used to provide fast relief of ocular symptoms in patients with AR.

**Recommendation: In patients with SAR under no ocular treatment, we suggest against starting OcAH (conditional recommendation based on low certainty of evidence) except for few days (7 or less) or as‐needed for very fast symptom relief. In patients with PAR under no treatment, we suggest using OcAH (conditional recommendation based on very low certainty of evidence)**. This recommendation is not focused on adding OcAH to a previous treatment.

- Considerations in children and adolescents: The recommendation is applicable to children and adolescents.
- Implementation considerations: None specific

**Change in recommendation from previous ARIA guidelines:** Previous ARIA guidelines suggested using OcAH over no treatment (conditional recommendation) in patients with SAR.

**Brief justification:** In PAR, the benefits of OcAH outweigh the risks for harms, but the same does not happen in SAR. See online supplement for more details.

**C. Questions with no change in recommendation directionality and/or strength in ARIA‐EAACI 2024–2025**.

**9. Should oral H_1_ ‐antihistamines vs. no treatment be used for the treatment of allergic rhinitis?**

**Link for the full EtD:** https://aria.med.up.pt/etd_oral‐06/

**Context:** OAH are one of the mainstays of the treatment of AR and are widely available.

**Recommendation: In patients with AR, we recommend using OAH over no treatment. (Strong recommendation based on moderate certainty of evidence)**

- Considerations in children and adolescents: The recommendation is applicable to children and adolescents.
- Implementation considerations: In LMIC, OAH in the WHO List of Essential Medicines and/or locally produced generics may be preferred.

**Brief justification:** OAH are overall efficacious, safe, cost‐effective and well‐accepted by patients. See online supplement for details.

**10. Should second‐generation oral H_1_ ‐antihistamines vs. first‐generation oral H_1_ ‐antihistamines be used for the treatment of allergic rhinitis?**

**Link for the full EtD:** https://aria.med.up.pt/etd_oral‐07/

**Context:** First‐generation OAHs are still frequently used in several countries, in part because of their low cost and over‐the‐counter availability. However, second‐generation OAHs are associated with improved safety profiles and reduced sedation.

**Recommendation: In patients with AR, we recommend using second‐generation OAH over first‐generation OAH (strong recommendation based on very low certainty of evidence)**.

- Considerations in children, adolescents and older adults: The recommendation is applicable to preschool‐aged, school‐aged children and older adults.
- Implementation considerations: See the implementation considerations for question number 1.

**Brief justification:** Second‐generation OAH are safer and associated with higher patient satisfaction. See online supplement for more details.

**11. Should intranasal H_1_ ‐antihistamines vs. oral H_1_ ‐antihistamines be used for the treatment of allergic rhinitis?**

**Link for the full EtD:** https://aria.med.up.pt/etd_oral‐08/

**Context:** While intranasal antihistamines (INAH) can have some benefits in terms of efficacy, OAH are more widely available and may have a higher acceptability.

**Recommendation: In patients with AR, we suggest either using INAH or OAH (conditional recommendation based on moderate certainty of evidence)**.

- Considerations in children and adolescents: The recommendation is applicable to preschool and school‐aged children
- Implementation considerations: None specific

**Brief justification:** INAH are associated with higher efficacy, but OAH present a lower risk of adverse events, higher affordability and acceptability. See online supplement for more details.

## 5 Conclusions

In ARIA‐EAACI 2024–2025, we formulated recommendations on eleven questions concerning oral or ocular treatments for AR. Overall, we suggest using INCS over second‐generation OAH, and second‐generation OAH over LTRA or OcAH. In addition, except in specific scenarios, we suggest against using LTRA or OcAH in untreated patients or as an addition to OAH. However, decisions on AR treatment should consider the clinical variability of the disease, patients' values and preferences, the affordability of treatment options, and planetary health considerations.

Questions on oral and ocular treatments had been previously addressed in past editions of the ARIA guidelines (see Box 2 and Table 4 for a comparison of recommendations of ARIA‐EAACI 2024–2025 with those of the ARIA 2010/2016 guidelines). However, four questions were addressed for the first time in ARIA‐EAACI 2024–2025, while, for four other questions, there was a change in the strength and/or directionality of recommendations.

**TABLE 4:** Comparison of the recommendations on oral and ocular treatments of the ARIA 2024–2025 and of the ARIA 2010/2016 guidelines.

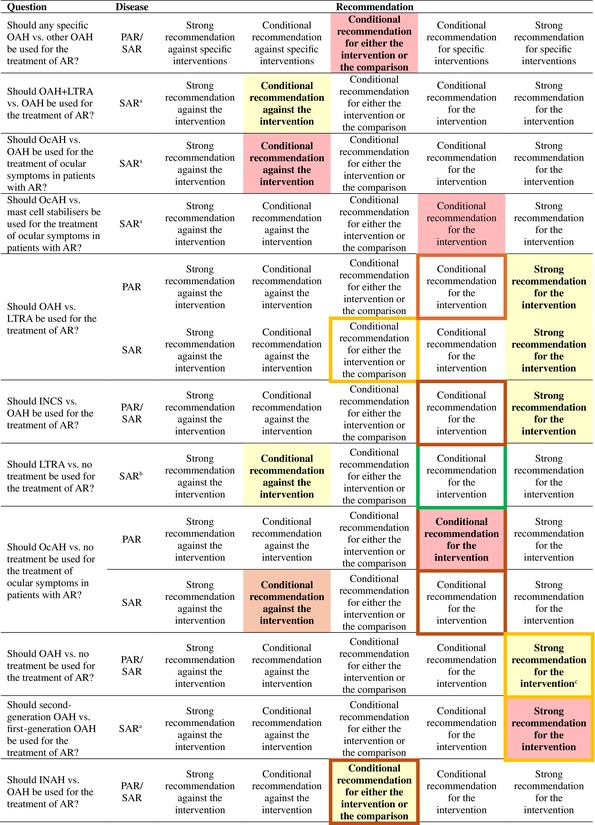

*Note:* Recommendations of the ARIA 2024–2025 guidelines are highlighted by a shade in a cell; recommendations of the ARIA 2010/2016 guidelines are highlighted by a frame in a cell. Shade/frame colour code: Green = High certainty of evidence; Yellow = Moderate certainty of evidence; Orange = Low certainty of evidence; Red = Very low certainty of evidence.

Abbreviations: AR, allergic rhinitis; INAH, intranasal antihistamine; INCS, intranasal corticosteroid; LTRA, oral leukotriene receptor antagonists; OAH, oral H_1_‐antihistamines; OcAH, ocular H_1_‐antihistamines; PAR, perennial allergic rhinitis; SAR, seasonal allergic rhinitis.

^a^ No evidence for PAR.

^b^ No evidence for PAR. In ARIA 2010, the recommendation was conditional against the use of LTRA in patients with PAR.

^c^ In ARIA 2010, the recommendation was strong for second‐generation OAH that do not cause sedation and do not interact with cytochrome P450, and conditional for second‐generation OAH that cause sedation and/or interact with cytochrome P450.

Considering all recommendations, second‐generation OAH are the preferred option among oral treatments for AR. Regarding individual second‐generation OAH, the identified evidence did not indicate that a specific medication should be recommended over all others. Instead, we suggest a shared decision‐making process considering criteria such as effectiveness, sedative effect, onset of action, affordability, availability and the patient's context and values. In these guidelines, we provide evidence on how the different second‐generation OAH compare in relation to the aforementioned aspects.

BOX 2 **Summary of what is new in the ARIA‐EAACI 2024–2025 guidelines in comparison to ARIA 2010/2016 guidelines.**

**Table:** 

*New questions*: Should any specific OAH vs. other OAH be used for the treatment of AR? ○ Recommendation: “In patients with AR, we suggest that the choice of specific OAH over others should be based on patients' preferences on efficacy and safety and on availability and affordability (conditional recommendation based on mostly very low certainty of evidence).” Should an OAH + LTRA vs. OAH be used for the treatment of AR? ○ Recommendation: “In patients with AR, we suggest using OAH over OAH+LTRA (conditional recommendation based on moderate certainty of evidence).” ^a^ Should OcAH vs. OAH be used for the treatment of ocular symptoms in patients with AR? ○ Recommendation: “In patients with AR, we suggest against using OcAH over OAH except for very fast relief of ocular symptoms (conditional recommendation based on very low certainty of evidence).” Should OcAH vs. ocular mast cell stabilisers be used for the treatment of ocular symptoms in patients with AR? ○ Recommendation: “In patients with AR, we suggest using OcAH over ocular mast cell stabilisers (conditional recommendation based on very low certainty of evidence).” *Questions with changed recommendation (in terms of directionality or strength)*: Should OAH vs. LTRA be used for the treatment of AR? ○ Recommendation changed from a *conditional recommendation in favour of either OAH or LTRA in seasonal AR* and a *conditional recommendation in favour of OAH in perennial AR* (2010/2016 guidelines) to a *strong recommendation in favour of OAH in both seasonal and perennial AR* (2024–2025 guidelines). ^a^ Should INCS vs. OAH be used for the treatment of AR? ○ Recommendation changed from a *conditional recommendation in favour of INCS* (2010/2016 guidelines) to a *strong recommendation in favour of INCS* (2024–2025 guidelines). Should LTRA vs. no treatment be used for the treatment of AR? ○ Recommendation changed from a *conditional recommendation in favour of LTRA in seasonal AR* and a *conditional recommendation against LTRA in perennial AR* (2010/2016 guidelines) to a *conditional recommendation against LTRA in both seasonal and perennial AR* (2024–2025 guidelines). ^a^ Should OcAH vs. no treatment be used for the treatment of ocular symptoms in patients with AR? ○ Recommendation changed from a *conditional recommendation in favour of OcAH* (2010/2016 guidelines) to a *conditional recommendation against OcAH in seasonal AR and a conditional recommendation in favour of OcAH in perennial AR*.

AR, allergic rhinitis; ARIA, allergic rhinitis and its impact on asthma; INCS, intranasal corticosteroids; LTRA, leukotriene receptor antagonists; OAH, oral H_1_‐antihistamines; OcAH, ocular H_1_‐antihistamines. ^a^Recommendations suggesting against the use of LTRA reflect, in part, the risk of rare neuropsychiatric adverse events.

By contrast, due to safety concerns and low effectiveness, LTRA should be restricted to specific situations, particularly for patients who are not well‐controlled with OAH and have a strong preference for oral treatments and/or comorbid asthma. Overall, the addition of an LTRA to an OAH is discouraged. The addition of oral decongestants to OAH has not been evaluated in these guidelines, as the corresponding question has not been prioritised, and a rapid literature review by our team did not find new, relevant RCTs. ARIA 2010 recommended against using OAH + oral decongestants versus OAH alone [8] (Table 5 lists ARIA 2010 recommendations on oral treatments for which questions were not prioritised in ARIA‐EAACI 2024–2025, namely oral decongestants and oral corticosteroids).

**TABLE 5:** ARIA 2010 guideline recommendations for oral medications which have not been evaluated in ARIA‐EAACI 2024–2025 guidelines (oral corticosteroids and oral decongestants).

Question	Recommendation
Should oral corticosteroids be used for treatment of AR in patients not responding to other therapy?	In patients with AR and moderate to severe nasal and/or ocular symptoms that are not controlled with other treatments, we suggest a short course of oral corticosteroids (conditional recommendation|very low‐quality evidence).
Should oral decongestant be used for treatment of AR?	In patients with AR, we suggest that clinicians do **not** administer and patients do **not** use oral decongestants regularly (conditional recommendation|low‐quality evidence).
Should a combination of oral decongestant and H_1_‐antihistamine versus oral H_1_‐antihistamine alone be used for treatment of AR?	In patients with AR, we suggest clinicians do **not** administer and patients do **not** use regularly a combination of oral H_1_‐antihistamine and an oral decongestant compared with oral H_1_‐antihistamine alone (conditional recommendation|moderate‐quality evidence).

When considering intranasal versus oral treatments, we recommend INCS (but not INAH) over OAH, as the former are more effective. On the other hand, OAH may be preferred to the use of topical ocular treatments. In fact, these guidelines suggest against using topical ocular treatments except for achieving a fast relief of eye symptoms, in which case, the use of OcAH is suggested over that of mast cell stabilisers.

Based on the first ARIA guidelines [6] and on the algorithm of ARIA 2016 [29], when initiating a treatment, physicians and pharmacists often propose a short course of OAH in mild rhinitis patients or in those with corticosteroid‐phobia [30, 31]. This approach does not contradict the ARIA‐EAACI 2024–2025 recommendations, and a new algorithm will be proposed (and subsequently tested) in a later report of the ARIA‐EAACI 2024–2025 guidelines.

Importantly, there are still knowledge gaps that would merit further research. Some of these gaps are common to those identified in the guidelines of intranasal treatments [18], including (i) lack of RCTs assessing patients with mild disease, (ii) lack of evidence stratified according to the presence of comorbid asthma, or according to patients' age group, sex and ethnicity, and (iii) lack of cost‐effectiveness studies or evidence on the planetary health of interventions. However, specific considerations should also be highlighted. In particular, there is less evidence informing recommendations on oral and ocular treatments in comparison to intranasal medications. Moreover, evidence on topical ocular treatments and on first‐generation OAH tended to be of particularly low quality, with a limited number of available eligible RCTs: in the case of topical ocular treatments, most RCTs evaluate short periods of time and/or only the immediate effect after conjunctival allergen challenge; for first‐generation OAH, most RCTs had been conducted prior to standardisation of outcome measurements. The scarce amount of evidence has resulted in downgrading the underlying certainty of evidence due to imprecision. Imprecision was also the certainty of evidence domain which was most frequently rated down in the NMA that informed the question comparing different individual OAH [24], pointing to the need for more and larger RCTs, particularly involving direct comparisons between active interventions.

As with the guidelines comparing intranasal treatments [18], we opted (i) not to present separate recommendations for SAR vs. PAR for most recommendations, and (ii) to refer to “perennial” or “seasonal AR” instead of “persistent” or “intermittent” AR (only a small amount of RCTs used the persistent/intermittent classification) [32, 33, 34]. In addition, we did not explore variations in the dosage or patterns of use (as‐needed versus regular) of treatments, as these questions will be addressed in future documents of the ARIA‐EAACI 2024–2025 guidelines.

These guidelines have limitations. For desirable and undesirable effects, evidence was mostly obtained from RCTs, in most cases with (i) an overrepresentation of patients with more severe AR, and (ii) a sample size and a follow‐up period that are too limited to detect serious but rare adverse events. This latter aspect is particularly relevant in the evaluation of LTRA. In addition, most included RCTs do not present stratified results for several relevant variables, including the presence of comorbid asthma. Finally, for OAH, several pivotal studies have evaluated only three nasal symptoms, excluding nasal congestion. The systematic reviews informing these guidelines have not considered these studies (even though this may result in a smaller number of included primary studies per OAH), (i) as nasal congestion is valued by patients as the most important symptom [20] and (ii) in order to ensure consistency within the different parts of the ARIA 2024–2025 guidelines.

There are also important strengths associated with ARIA‐EAACI 2024–2025. We have followed the GRADE approach, using EtDs to develop recommendations. In addition, we have used several approaches to formulate guideline questions and considered different data sources. The ARIA‐EAACI 2024–2025 guidelines have a global scope and recommendations have implementation considerations for LMIC. In addition to these considerations, the links of the EtD display maps informing about the most affordable medications in different countries. Finally, we have conducted several systematic reviews and meta‐analyses to provide updated evidence on the desirable and undesirable effects of interventions.

In conclusion, this article of the ARIA‐EAACI 2024–2025 guidelines focuses on oral and ocular treatments for the management of AR. It has been developed following the GRADE approach and considering evidence from a comprehensive set of evidence from multiple sources, such as systematic reviews of RCTs, mHealth data and a survey of experts.

## Author Contributions

Bernardo Sousa‐Pinto, Jean Bousquet, Holger J. Schünemann and Torsten Zuberbier were responsible for the coordination of the project (as members of the ARIA 2024–2025 guidelines steering committee) and contributed to the methodology (including evidence synthesis and analysis), the discussion of the evidence and the drafting of recommendations (as members of the ARIA 2024–2025 guideline panel) and writing the manuscript. Rafael José Vieira and Antonio Bognanni contributed to the methodology (including evidence synthesis and analysis), discussion of the evidence and drafting of recommendations (as members of the ARIA 2024–2025 guideline panel) and writing the manuscript. Arunas Valiulis, Sian Williams, Anna Bedbrook, Maria Jose Torres, G. Walter Canonica, Leticia de las Vecillas, Mark S. Dykewicz, Cristina Jacomelli, Ludger Klimek, Lucas Leemann, Olga Lourenço, Nikolaos G. Papadopoulos, Ana Margarida Pereira, Marine Savouré, Sanna K. Toppila‐Salmi, Maria Teresa Ventura, Juan José Yepes‐Nuñez, Elena Azzolini, Gilles Louis, Elena Parmelli and Jaron Zuberbier contributed to the discussion of the evidence and drafting of recommendations (as members of the ARIA 2024–2025 guideline panel) and writing the manuscript. Rita Amaral, Sara Gil‐Mata, Manuel Marques‐Cruz, Ewa Borowiack, Raquel Albuquerque Costa, Henrique Pereira, Renato Ferreira‐da‐Silva, Despo Ierodiakonou, Justyna Litynska, Inês Ribeiro‐Vaz, Ewelina Sadowska, Tuuli Thomander and João A. Fonseca contributed to the methodology (including evidence synthesis and analysis) and revising and editing the manuscript. All other authors were part of the international panel revising the recommendations, contributing to the guidelines by providing feedback to the recommendations and revising and editing the manuscript.

## Funding

The work of this study has been funded within the context of the Allergic Rhinitis and its Impact on Asthma (ARIA) group.

## Disclosure

Disclaimer: Dr. Alkis Togias' co‐authorship of this report does not constitute endorsement by the National Institute of Allergy and Infectious Diseases, the National Institutes of Health, or any other Agency of the United States Government.

## Conflicts of Interest

Jean Bousquet reports personal fees from Cipla, Menarini, Mylan, Novartis, Purina, Sanofi‐Aventis, Teva, Noucor, other from KYomed‐Innov, other from Mask‐air‐SAS, outside the submitted work. Oliver Pfaar reports grants and personal fees from ALK‐Abelló, grants and personal fees from Allergopharma, grants and personal fees from Stallergenes Greer, grants and personal fees from HAL Allergy Holding B.V./HAL Allergie GmbH, grants and personal fees from Bencard Allergie GmbH/Allergy Therapeutics, grants and personal fees from Laboratorios LETI/LETI Pharma, grants and personal fees from GlaxoSmithKline, personal fees from ROXALL Medizin, personal fees from Novartis, grants and personal fees from Sanofi‐Aventis and Sanofi‐Genzyme, personal fees from Med Update Europe GmbH, personal fees from streamedup! GmbH, personal fees from Pohl‐Boskamp, grants from Inmunotek S.L., personal fees from John Wiley and Sons, AS, personal fees from Paul‐Martini‐Stiftung (PMS), personal fees from Regeneron Pharmaceuticals Inc., personal fees from RG Aerztefortbildung, personal fees from Institut für Disease Management, personal fees from Springer GmbH, grants and personal fees from AstraZeneca, personal fees from IQVIA Commercial, personal fees from Ingress Health, personal fees from Wort&Bild Verlag, personal fees from Verlag ME, personal fees from Procter&Gamble, personal fees from ALTAMIRA, personal fees from Meinhardt Congress GmbH, personal fees from Deutsche Forschungsgemeinschaft, personal fees from Thieme, grants from Deutsche AllergieLiga e.V., personal fees from AeDA, personal fees from Alfried‐Krupp Krankenhaus, personal fees from Red Maple Trials Inc., personal fees from Königlich Dänisches Generalkonsulat, personal fees from Medizinische Hochschule Hannover, personal fees from ECM Expro&Conference Management, personal fees from Technical University Dresden, grants and personal fees from Lilly, personal fees from Japanese Society of Allergy, personal fees from Forum für Medizinische Fortbildung, personal fees from Dustri‐Verlag, personal fees from Pneumolive, grants and personal fees from ASIT Biotech, personal fees from LOFARMA, personal fees from Paul‐Ehrlich‐Institut, personal fees from Almirall, from BREAZY Health, outside the submitted work; and Vice President and member of EAACI Excom, member of ext. board of directors DGAKI; coordinator, main‐ or co‐author of different position papers and guidelines in rhinology, allergology and allergen‐immunotherapy; Editor‐in‐Chief (EIC) of Clinical Translational Allergy (CTA), Associate Editor (AE) of Allergy. Roland Buhl reports personal fees from AstraZeneca, Berlin‐Chemie, Celltrion, Chiesi, Cipla, Sanofi and Teva, as well as grants to Mainz University Hospital and personal fees from Boehringer Ingelheim, GlaxoSmithKline, Novartis and Roche outside the submitted work. Michael Hyland reports grants from GSK, grants from AstraZeneca, and from null, outside the submitted work. Maciej Kupczyk reports personal fees from Adamed, personal fees from Astra Zeneca, personal fees from Abbvie, personal fees from Berlin Chemie, personal fees from Chiesi, personal fees from Emma, personal fees from Lekam, personal fees from Aurovitas, personal fees from Novartis, personal fees from Teva, personal fees from GSK, personal fees from Sanofi, personal fees from HVD, personal fees from Zentiva, personal fees from Celon Pharma, personal fees from HAL Allergy, personal fees from Glenmark, personal fees from Stada, outside the submitted work. Robert N. Naclerio reports personal fees from Lyra, personal fees from Sanofi, outside the submitted work. Sven Becker reports personal fees from Sanofi, personal fees and non‐financial support from AstraZeneca, personal fees and non‐financial support from GSK, personal fees and non‐financial support from Bencard Allergie, personal fees from Allergy Therapeutics, personal fees from ALK Abelló, personal fees and non‐financial support from HAL Allergie, grants from BMBF, outside the submitted work. Dana Wallace reports: I was one of the primary authors on the most recent JTFPP Rhinitis PP. However, I don't believe that this influenced my review and recommendation to submit this paper for publication. Juan Carlos Ivancevich reports personal fees from Laboratorios Casasco Argentina, personal fees from World Allergy Organisation, personal fees from Global Asthma Association (Interasma) outside the submitted work. Margitta Worm reports other from AbbVie Deutschland GmbH & Co. KG, other from Aimmune Therapeutics UK Limited, other from ALK‐Abelló Arzneimittel GmbH, other from Allergopharma GmbH & Co KG, other from Almirall Hermal GmbH, other from Amgen GmbH, other from AstraZenceca GmbH, other from Bayer AG, other from Bencard Allergy GmbH, other from Bioprojet Pharma, other from Boehringer Ingelheim Pharma GmbH &Co.KG, other from Bristol Myers Squibb GmbH & Co. KGaA, other from Galderma Laboratorium GmbH, other from Glaxo Smith Kline GmbH & Co. KG, other from Infectopharm Arzneimittel und Consilium GmbH, other from LEO Pharma GmbH, other from Lilly Deutschland GmbH, other from Mylan Germany GmbH (A Viatris Company), other from Novartis AG, other from Octapharm AG, other from Pfizer Pharma GmbH, other from Sanofi‐Aventis Deutschland GmbH/Genzyme Europe B. B.; other from Stallergenes GmbH, outside the submitted work. Elina Toskala reports other from Sanofi, grants and other from GSK, grants and other from Aerin, other from Medtronic, outside the submitted work. Michael S. Blaiss reports personal fees from Opelia, personal fees from Bayer outside the submitted work. Miloš Jesenak reports speaker's fee and honoraria from BerlinChemie Menarini, Chiesi, MSD, GlaxoSmithKlime, Abbvie, AsrtaZeneca, Viatris, ALK, Stallergenes‐Greer, Glenmark, Takeda, Novartis, Zentiva; travel grants from Zentiva, Takeda, Novartis, ALK, Stallergenes‐Greer, Abbvie; and honoraria for serving as a member of Advisory Board for AstraZeneca, Viatris, Stallergenes‐Greer, ALK, Novartis, Chiesi, CSL Behring, Abbvie and Takeda, outside the submitted work. Joaquin Sastre reports grants and personal fees from SANOFI, personal fees from GSK, personal fees from NOVARTIS, personal fees from ASTRA ZENECA, personal fees from MUNDIPHARMA, personal fees from FAES FARMA, outside the submitted work. Thomas Casale reports grants and personal fees from ELI LILLY, outside the submitted work. Justyna Litynska reports others from Fraunhofer Institute, during the conduct of the study. Tomohisa Iinuma reports grants from Sanofi, grants from Mochida, outside the submitted work. Tari Haahtela reports speaker's fee from ALK Nordic outside the submitted work. Désirée E. Larenas‐Linnemann reports personal fees from ALK, Armstrong, Astrazeneca national and global, Chiesi, Grunenthal, GSK national and global, Viatris, Megalabs Ecuador, Naos, Novartis, Pfizer, Sanofi, Siegfried, Syneos Health, grants from Abbvie, Lilly, Sanofi, Astrazeneca, Pfizer, Novartis, Pulmonair, GSK, Chiesi, Biopharma, outside the submitted work; and Editor in chief of Immune System (Karger)Ðember of asthma committee ACAAIÐubgroup chair of allergen immunotherapy Practice parameter update JTF AAAAI/ACAAI 2024Ðember of allergen immunotherapy committee AAAAIÜhair of allergen immunotherapy committee CMICAÐember of allergic asthma task force EAACI. Brigita Gradauskiene reports personal fees from Viatris, personal fees from Berlin‐Chemie Menarini, grants and personal fees from AstraZeneca, personal fees from Mylan Healthcare, personal fees from AbbVie, outside the submitted work. G. Walter Canonica reports having received research grants as well as being lecturer or having received advisory board fees from A.Menarini, Anallergo, Allergy Therapeutics, AstraZeneca, Celltrion, Chiesi Farmaceutici, Faes, Firma, Genentech, GrandPharma, Guidotti Glaxo Smith Kline, Hal Allergy, Innovacaremd, Novartis, OmPharma, RedMaple, Sanofi‐Aventis, Sanofi‐Genzyme, Stallergenes‐Greer, Uriach Pharma, outside the submitted work. Daiju Sakurai reports grants and personal fees from Torii, personal fees from Tanabe Mitsubishi, personal fees from Shionogi, grants and personal fees from Taiho, grants and personal fees from Kyorin, personal fees from Meiji seika pharma, personal fees from Thermo fisher scientific diagnostics, personal fees from Tsumura, personal fees from Sanofi, grants and personal fees from Astra Zeneca, personal fees from Hisamitsu, outside the submitted work. Sanna K. Toppila‐Salmi reports grants and other from GSK, grants and other from Sanofi, other from AstraZeneca, other from ALK‐Abelló, other from OrionPharma outside the submitted work. Marianne van Hage reports personal fees from Thermo Fisher Scientific, outside the submitted work. Giovanni Paoletti reports personal fees from GSK, personal fees from LoFarma, personal fees from Astrazeneca, personal fees from Sanofi outside the submitted work. Oscar Palomares reports having received research grants from MINECO, Ministerio de Ciencia e Innovación, CAM, Inmunotek S.L., Novartis and AstraZeneca and fees for giving scientific lectures or participation in Advisory Boards from AstraZeneca, Pfizer, GlaxoSmithKline, Inmunotek S.L., Novartis, Regeneron and Sanofi, outside the submitted work. Jan Romantowski reports personal fees from GSK, personal fees from Sanofi, personal fees from AstraZeneca outside the submitted work. Nikolaos G. Papadopoulos reports personal fees from Nestlé Nutrition Institute, personal fees from Abbott Nutrition, grants from Numil Hellas SA, grants from VIANEX, personal fees from GSK, personal fees from HAL Allergy Holding B.V, personal fees from Menarini International Operations Luxembourg SA, personal fees from Regeneron Pharmaceuticals Inc., personal fees from Berlin—Chemie AG, personal fees from DBV Technologies SA, grants from Vibrant America, personal fees from Hyproca Nutrition USA INC, personal fees from Danone Trading Medical B.V., personal fees from Med Maps srl, outside the submitted work. Helga Kraxner reports Speaker's fee and congress support from Sanofi, from Viatris, from Berlin‐Chemie, from Ewopharma, from AstraZeneca; Advisory Board membership of Sanofi, of AstraZeneca, of Berlin‐Chemie. Dermot Ryan reports personal fees from Thermo‐Fisher, personal fees from Menarini, personal fees from Viatris, outside the submitted work. Marcin Moniuszko reports having received in the past personal fees, non‐financial support and other from Berlin‐Chemie/Menarini, personal fees, non‐financial support and other from Astra Zeneca, personal fees, non‐financial support and other from GlaxoSmithKline, personal fees, non‐financial support and other from Novartis, personal fees, non‐financial support and other from Chiesi, personal fees, non‐financial support and other from Celon Pharma, personal fees, non‐financial support and other from Takeda, personal fees, non‐financial support and other from Polfarmex, personal fees, non‐financial support and other from CSL Behring, personal fees and non‐financial support from Glenmark Pharmaceuticals, personal fees, non‐financial support and other from Sanofi, personal fees, non‐financial support and other from Teva, outside the submitted work. Ana Todo‐Bom reports personal fees from GSK, personal fees from Leti pharma, grants from AbbVie, personal fees from Amirall, grants and personal fees from Astra Zeneca, grants from Sanofi, outside the submitted work. Yoshitaka Okamoto reports personal fees from Torii pharmaceutical Co. Ltd., personal fees from Tanabe‐Mitsubishi Pharmaceutical Co. Ltd., personal fees from Kirin Holdings Co. Ltd., personal fees from Shionogi Co. Ltd., personal fees from Stallergenes‐Greer, personal fees from Diichi‐Sankyo, outside the submitted work. Joaquim Mullol reports personal fees and other from SANOFI‐GENZYME & REGENERON, personal fees and other from NOVARTIS, grants, personal fees and other from VIATRIS/MEDA Pharma, grants and personal fees from NOUCOR/URIACH Group, personal fees from Menarini, personal fees from UCB, personal fees and other from AstraZeneca, grants, personal fees and other from GSK, personal fees from MSD, personal fees and other from Lilly, personal fees and other from GLENMARK, personal fees from ALMIRALL, outside the submitted work. Heidi Olze received fees from F. Hoffmann‐La Roche Ltd., Sanofi‐Aventis Deutschland GmbH, AstraZeneca GmbH, GlaxoSmithKline GmbH & Co. KG and Novartis outside of the present work. Constantinos Pitsios reports he is a member of EAACI ExCom, Guest Editor for special issues of Medicina and the International Journal of Molecular Science, and a member of the Editorial board of Clinical and Molecular Allergy. M. Wagenmann reports personal fees from Allergopharma, personal fees from ALK‐Abello, grants and personal fees from AstraZeneca, personal fees from CSL Behring, grants and personal fees from GSK, personal fees from HAL, personal fees from Leti Pharma, personal fees from MSD, personal fees from Novartis, grants and personal fees from Regeneron, grants and personal fees from Sanofi, personal fees from Stallergenes, grants from Takeda, outside the submitted work. Janet M. Davies reports grants from Australian National Health and Medical Research Council, grants from Australian Medical Research Future Fund, grants from Australian Research Council, grants from National Allergy Centre of Excellence, grants from Bayer Health Care, outside the submitted work; In addition, Dr. Janet M. Davies has a patent Grass pollen allergen diagnostics issued and QUT has received in kind provision of materials and services for research from non‐financial support from Abionic SA Switzerland, Swisens SA Switzerland, Kenelec Australia, ThermoFisher Sweden and Sullivan Nicolaides Pathology Australia, outside the submitted work. Philippe Devillier reports personal fees from ALK‐Abello, personal fees from Chiesi, personal fees and non‐financial support from Astra Zeneca, personal fees from GlaxoSmithKline, personal fees from Viatris, personal fees from Menarini, personal fees and non‐financial support from Stallergenes, personal fees from Congrès de Pneumologie de Langue Française, non‐financial support from Congrès Français d'Allergologie outside the submitted work. Jaime Correia de Sousa reports other from Boheringer Ingelheim, personal fees and other from GSK, grants, personal fees and other from AstraZeneca, non‐financial support from Bial, non‐financial support from Mundipharma, personal fees and other from Sanofi, from Novartis, personal fees from MSD, personal fees from Medinfar, outside the submitted work. Nicolas Roche reports grants and personal fees from GSK, personal fees from AstraZeneca, grants and personal fees from Chiesi, grants and personal fees from Pfizer, personal fees from Sanofi, personal fees from Zambon, personal fees from MSD, personal fees from Austral, personal fees from Biosency, outside the submitted work. Charlotte Suppli Ulrik reports grants and personal fees from AZ, personal fees from GSK, personal fees from Chiesi, personal fees and non‐financial support from Orion Pharma, grants and personal fees from Boehringer Ingelheim, personal fees from Sanofi, personal fees from Pfizer, personal fees from Novartis, personal fees from IQVIA, personal fees from TFF Pharmaceuticals, personal fees from Berlin Chemie, personal fees from Hikma Pharmaceuticals, personal fees from TEVA, personal fees from Roche, personal fees from Regeneron, personal fees from Novo Nordisk, personal fees from Covis Pharma, personal fees from Takeda, outside the submitted work. Jonathan A. Bernstein reports grants from Allergy Therapeutics, grants from ALK, outside the submitted work. I am also on the JTF for the AAAAI/ACAAI and co‐author on the Rhinitis guidelines. I am Chairman of the AAAAI Foundation and a member of the WAO board of directors. Maria Jose Torres reports personal fees from Leti Laboratories, personal fees from Aimmune Therapeutics, personal fees from Diater Laboratories, grants from European Commission, grants from ISCIII, grants from SEAIC outside the submitted work. Mattia Giovannini reports personal fees from Sanofi, Thermo Fisher Scientific, outside the submitted work. Victoria Cardona reports personal fees from GSK, personal fees from Organon, personal fees from Roxall, personal fees from Viatris, personal fees from Allergy Therapeutics, outside the submitted work. Dr. Pascal Werminghaus reports personal fees from Astrazeneca, personal fees from Allergy Therapeutic, personal fees from GSK, personal fees from Sanofi, personal fees from Stallergenes, personal fees from Celltrion outside the submitted work. Ioanna Tsiligianni reports grants from Chiesi, GSK Hellas, Menarini, Astra Zeneca Greece, outside the submitted work. Lorenzo Cecchi reports personal fees from Astra Zeneca, personal fees from Menarini, personal fees from Sanofi, personal fees from GSK, personal fees from Firma, personal fees from Thermofisher, personal fees from Novartis, personal fees from Chiesi, outside the submitted work. Graham Roberts reports non‐financial support from ThermoFisher, outside the submitted work. Paul Keith reports personal fees from ALK, personal fees from Bausch, personal fees from Canadian Agency for Drugs and Technologies in Health, personal fees from Bayer, personal fees from GSK, personal fees from Medexus, personal fees from Novartis, personal fees from Sanofi, outside the submitted work. Sophia Neisinger reports personal fees from Novartis, personal fees from Biocryst, personal fees from Celltrion, personal fees from Sanofi outside the submitted work. Markus Ollert reports personal fees from Allergy Therapeutics/Bencard GmbH, personal fees from Thermo Fisher Scientific, personal fees from Hycor Diagnostics, personal fees from Medice/Theralution GmbH, personal fees from Streamed‐Up GmbH, personal fees from Stallergenes‐Greer, outside the submitted work; in addition, Dr. Ollert has a patent WO2019076477A1 pending to Tolerogenics Sarl and Scientific Co‐Founder, Tolerogenics Sarl. Enrico Compalati reports personal fees from Lofarma spa during the conduct of the study. Tuuli Thomander reports grants from The Finnish ORL‐HNS Foundation, grants from The Research Foundation of the Pulmonary Diseases, grants from The Maud Kuistila Memorial Foundation, grants from The Foundation of the Finnish Anti‐Tuberculosis Association, during the conduct of the study. Sian Williams reports that like many people, she has allergic rhinitis with her own personal preference for treatment, and this therefore required reflexivity when reviewing the research. As a result, she does not believe this has affected her judgement; in fact, it has informed what she will do in the future! Luís Taborda‐Barata reports personal fees from LETI, personal fees from Sanofi, outside the submitted work. Alvaro A. Cruz reports grants, personal fees and non‐financial support from AstraZeneca, personal fees from Chiesi, personal fees from GSK, grants and personal fees from Eurofarma, personal fees from Farmoquimica, personal fees from Glennmark, grants and personal fees from Sanofi, and grants from EMS outside the submitted work. Holger J. Schünemann reports developed guidelines on Allergic Rhinitis in Asthma (ARIA) and his academic institution received research funding for it. The other authors declare no conflicts of interest outside the submitted paper.

## Supporting information


**Data S1:** all70305‐sup‐0001‐DataS1.docx.

## Data Availability

The authors have nothing to report.
