# Supplementary material for: Allergic Rhinitis and Its Impact on Asthma (ARIA)‐EAACI Guidelines—2024–2025 Revision: Part II—Guidelines on Oral and Ocular Treatments
Source: Allergy. 2026 Mar 24;81(6):1947–70. doi: 10.1111/all.70305 (PMC13256267; doi:10.1111/all.70305)
Supplement: Supplementary file 1 — Data S1: all70305‐sup‐0001‐DataS1.docx. [file ALL-81-1947-s001.docx]

**Online supplement of the Allergic Rhinitis and its Impact on Asthma (ARIA)-EAACI guidelines – 2024-2025 revision: Part II – Guidelines on oral and ocular treatments**

**Brief description of the methodology to develop the ARIA-EAACI guidelines**

**Questions and outcomes of interest**

In Allergic Rhinitis and its Impact on Asthma (ARIA) – European Academy of Allergy and Clinical Immunology (EAACI) 2024-2025, four distinct approaches were employed to develop clinical guideline questions: (i) reviewing questions addressed in previous ARIA guidelines and US Practice Parameters, (ii) surveying of ARIA panel members [healthcare professional-centred questions], (iii) identification of questions emerging from MASK-air^®^ studies, and (iv) use of artificial intelligence (AI) to support the development of guideline questions^1^ [patient-centred questions]. The resulting questions were then subject to the Grading of Recommendations, Assessment, Development and Evaluation (GRADE) formal process of prioritisation^2^ using GRADEpro^3^.

For each guideline question, the following set of outcomes were considered (prioritised using GRADE formal processes^2^): nasal symptoms, ocular symptoms, quality of life, total symptoms, serious adverse events, and occurrence of any adverse event. Therefore, efficacy outcomes comprised nasal, ocular and total symptoms, as well as quality of life. Safety outcomes comprised any/total adverse events and serious adverse events.

**Evidence review and development of recommendations**

For each question, we gathered evidence on the various criteria of the evidence-to-decision (EtD) framework, a systematic and transparent approach designed to support the formulation of recommendations^4,5^. The EtD includes 12 standard criteria: priority, desirable and undesirable effects, certainty of evidence, values and preferences, balance of effects, resources required (and corresponding certainty of evidence), cost-effectiveness, equity, acceptability, and feasibility. In addition, the ARIA 2024-2025 guidelines included a 13^th^ criterion: planetary health^6,7^.

Evidence on desirable and undesirable effects of oral medications was obtained by conducting two systematic reviews (SRs) of randomized controlled trials (RCTs): (i) comparisons among oral medications in adults^8^, and (ii) comparisons among oral medications in children (Thomander et al., Under review). For ocular antihistamines (OcAH), we relied on two previously published SRs^9,10^. In addition, evidence on undesirable effects was complemented by an analysis of pharmacovigilance data from VigiBase, the World Health Organization’s (WHO) global database of adverse events^11^.

To gather evidence on patients’ values, we conducted a SR of the literature^12^. For the remaining criteria, we performed non-systematic evidence reviews, which were complemented by evidence from other sources. Specifically, we conducted a survey among ARIA experts to assess the availability and costs of various allergic rhinitis (AR) medications (in preparation). We also analysed MASK-air^®^ direct patient data to obtain information on treatment acceptability (in particular, adherence, satisfaction, and use of co-medication) and to estimate indirect costs associated with work productivity loss. The WHO List of Essential Medicines was consulted to inform judgements on equity^13^.

Considering that the ARIA 2024-2025 panel had already held regular online meetings to reach judgements on the guideline questions for intranasal treatments and that the process had been highly consensual, with minimal disagreement, the process was streamlined for the EtDs on oral and ocular treatments. For these, the ARIA 2024-2025 methodology team pre-filled the EtDs with proposed judgements and circulated them to all panel members via email for feedback and discussion. Revisions were made iteratively until consensus was reached on both the judgements and the final recommendations. If consensus was not reached, a formal voting process was set with all voting members (i.e., members with no conflicts of interest). Recommendations were worded following the GRADE working group guidance (see “How to use these guidelines” section)^14^. In addition, for each recommendation, we present (i) considerations for pre-school and school-aged children and adolescents (and, if evidence is available, other special populations, such as patients with asthma), and (ii) implementation considerations. The latter include, among others, aspects related to the application of recommendations in low- and middle-income countries, or concerns with specific adverse events.

The final form of guideline recommendations and their wording, as well as the final guideline document, have been reviewed and approved by all panel members.

**Brief justification of guideline questions**

**Brief justification of question 7: Should leukotriene receptor antagonists vs. no treatment be used for the treatment of allergic rhinitis?**

- Efficacy and safety:
  - A meta-analysis assessing the effect of leukotriene receptor antagonists (LTRA) on nasal symptoms compared to placebo included eight randomized controlled trials (RCTs) in seasonal allergic rhinitis (SAR) (data in the EtD link). LTRA were associated with an improvement in nasal symptoms (65% probability of a small but meaningful difference). For perennial allergic rhinitis (PAR), only one RCT was identified, and the effect of LTRA was mostly trivial.
  - For ocular symptoms, we included six RCTs in SAR. LTRA were associated with an improvement in ocular symptoms (48% probability of a small but meaningful difference). No studies were identified for PAR.
  - Regarding rhinoconjunctivitis quality of life questionnaire (RQLQ), six RCTs concerned patients with SAR. LTRA were associated with an improvement in RQLQ scores (89% probability of a small but meaningful difference). For PAR, two RCTs were identified, and LTRA were associated with a 51% probability of a meaningful improvement.
  - In a systematic review of RCTs, we found no significant difference in the frequency of adverse events between LTRA and placebo in patients with SAR. No RCTs reporting on adverse events were identified for PAR. However, observational studies and pharmacovigilance reports have raised concerns about potential neuropsychiatric effects linked to LTRA, including depression, suicidal ideation, and insomnia. These concerns led the Food and Drug Administration (FDA) to issue a black box warning^15^.
- Resources required, cost-effectiveness and equity: A survey of Allergic Rhinitis and its Impact on Asthma (ARIA) experts from 51 countries indicated that LTRA, particularly montelukast, are widely available. The yearly cost of LTRA ranged from 8.9 to 2256.5 US Dollars Purchase Power Parity (USD PPP), depending on the country. These costs are notably lower than the productivity losses associated with poorly controlled AR, which may exceed 500 USD PPP per week in Western Europe. No cost-effectiveness studies comparing LTRA to no treatment were identified. LTRA are not included in the World Health Organization (WHO) List of Essential Medicines.
- Acceptability and feasibility: Evidence from MASK-air data suggests high patient satisfaction with LTRA monotherapy (median visual analogue scale [VAS] = 94/100), though few observations were identified (*N*=337). LTRA are frequently used with other medications (83.0% of days) and their onset of action is less than two hours.
- Planetary health: The manufacturer life cycle assessment internal report indicated that montelukast was responsible for 0.23-0.47 kg CO_2_e/product^16^. The impact of LTRA on production, packaging and transport may be offset by the reduced emissions that a better level of allergic rhinitis (AR) control may entail (i.e., reduced need for healthcare visits and over-the-counter medications).

**Brief justification of question 8: Should ocular H_1_-antihistamines vs. no treatment be used for the treatment of ocular symptoms in patients with allergic rhinitis?**

- Efficacy and safety:
  - Primary studies did not allow estimating meta-analytical measures. In SAR, identified studies suggested trivial differences when comparing the improvement of ocular symptoms between ocular antihistamines (OcAH) and placebo. By contrast, in PAR, a clinically meaningful effect favouring OcAH was observed.
  - Regarding safety, OcAH were associated with a higher risk of adverse events compared with placebo. In SAR, the meta-analytical results suggested the difference to be trivial to small (but meaningful). In perennial allergic rhinitis, the meta-analytical results suggested the difference to be trivial to moderate. Serious adverse events were not reported in the identified trials.
- Resources required, cost-effectiveness and equity: A survey of ARIA experts from 51 countries indicated that OcAH are widely available. The yearly cost of OcAH ranged from 13.2 to 947.3 USD PPP, depending on the country. No cost-effectiveness studies comparing OcAH to no treatment were identified. OcAH are not included in the WHO List of Essential Medicines.
- Acceptability and feasibility: Evidence from MASK-air data suggests high patient satisfaction with OcAH monotherapy (median VAS = 86/100). OcAH are frequently used with other medications (in 88.7% of days). Regarding their onset of action, several studies that performed conjunctival allergen challenges reported that OcAH can be associated with a relief of ocular symptoms within three minutes.
- Planetary health: No specific quantitative evidence was found in terms of the impact of OcAH on Planetary Health. OcAH frequently come in small plastic vials with short expiry time windows – their occasional use may result in relevant waste.

**Brief justification of question 9: Should oral H_1_-antihistamines vs. no treatment be used for the treatment of allergic rhinitis?**

- Efficacy and safety:
  - A network meta-analysis (NMA) (data in the EtD link) found that oral antihistamines (OAH) improve nasal symptoms compared to placebo in both SAR and PAR, with a 100% probability of achieving a small but meaningful improvement.
  - OAH also improve ocular symptoms compared to placebo in SAR, with a 91% probability of a small but meaningful benefit. No evidence was available for PAR.
  - Regarding RQLQ, OAH improve outcomes compared to placebo in both SAR and PAR, respectively displaying an 87% and 78% probability of a small but meaningful benefit.
  - Adverse events and serious adverse events occurred at similar frequencies with OAH and placebo, with only trivial differences observed. Most adverse events were mild and self-limited.
- Resources required, cost-effectiveness and equity: A survey of ARIA experts suggested that OAH are widely available (being available in all respondents’ countries) and that the costs of OAH vary widely across countries. However, these weekly costs appear to be considerably lower than the productivity losses observed in patients with poorly controlled AR, which may exceed 500 USD PPP per week in Western European countries. No recent cost-effectiveness studies comparing OAHs to no treatment were identified. Cetirizine, fexofenadine and loratadine are in the WHO List of Essential Medicines.
- Acceptability and feasibility: MASK-air^®^ data suggest that OAH are associated with high treatment satisfaction and lower odds of being used in co-medication compared to other commonly used drug classes. Medication adherence during the pollen season appears to be higher than that observed for intranasal antihistamines (INAH) and similar to that of intranasal corticosteroids (INCS). OAH display a relatively fast onset of action, with symptom relief typically beginning within 1 to 2 hours.
- Planetary health: No specific evidence was found in terms of the impact of OAH on Planetary Health. The impact of OAH on production, packaging and transport may be offset by the reduced emissions that a better level of AR control may entail (i.e., reduced need for healthcare visits and over-the-counter medications).

**Brief justification of question 10: Should second-generation oral H_1_-antihistamines vs. first-generation oral H_1_-antihistamines be used for the treatment of allergic rhinitis?**

- Efficacy and safety:
  - A NMA found very limited evidence comparing second-generation and first-generation OAHs for nasal symptom improvement in AR^8^ (data in the EtD link). Among 37 RCTs in SAR evaluating the total nasal symptom score computed based on four symptoms, only one directly compared a first-generation OAH (dexchlorpheniramine) with a second-generation OAH (terfenadine), showing greater improvement with the first-generation drug, with a 97% probability of a non-trivial difference. Other studies, not included in the analysis due to differences in outcome reporting, reported mixed results. No studies were found comparing OAH of the two generations in PAR.
  - For ocular symptoms, no evidence was found.
  - Regarding RQLQ, among the 22 RCTs in SAR, only one assessed a first-generation OAH (chlorpheniramine), showing no differences in RQLQ improvement compared to second-generation OAHs (64% probability of a trivial difference). No studies were identified for PAR.
  - Regarding safety, the same NMA found that second-generation OAHs were associated with a lower frequency of adverse events compared to first-generation. The included trials did not provide data on serious adverse events, but evidence from other reviews suggests that first-generation OAHs may impair cognitive and driving performance and are associated with higher self-reported sedation^17,18^. Pharmacovigilance data further support a more favourable safety profile for second-generation OAHs, with important adverse events reported less frequently than for first-generation agents.
- Resources required, cost-effectiveness and equity: We did not identify any studies that satisfactorily addressed the comparison of new- vs. first-generation OAH in terms of resources or equity. Estimates using unadjusted MASK-air^®^ data suggest that second-generation OAHs may be cost-effective. The only OAH on the WHO List of Essential Medicines are second-generation OAHs (cetirizine, fexofenadine and loratadine).
- Acceptability and feasibility: Evidence from an international survey suggests that treatment satisfaction is generally higher for second-generation OAHs. MASK-air^®^ data further support this finding: second-generation OAHs are associated with higher treatment satisfaction and better adherence compared to first-generation OAHs. First-generation OAHs are also more frequently used in co-medication than second-generation agents.
- Planetary health: No specific evidence was found in terms of comparative impact on planetary health.

**Brief justification of question 11: Should intranasal H_1_-antihistamines vs. oral H_1_-antihistamines be used for the treatment of allergic rhinitis?**

- Efficacy and safety:
  - A pairwise meta-analysis comparing INAH *versus* OAH on nasal symptoms included three RCTs in SAR^19^. INAH were associated with an improvement in nasal symptoms (44% probability of resulting in a small but meaningful difference). Consistent results were observed when INAH and OAH were compared in a NMA (data in the EtD link). For PAR, no RCT were identified directly comparing INAH *versus* OAH. Indirect comparisons from a NMA revealed that OAH tended to result in greater improvement in nasal symptoms compared to INAH, even though the difference did not reach statistical significance (data in the EtD link).
  - For ocular symptoms in patients with SAR, no studies were identified directly comparing INCS *versus* OAH. A NMA revealed that INAH was associated with a high improvement in ocular symptoms compared to OAH, but the difference was trivial (data in the EtD link). No evidence was obtained for PAR.
  - Regarding RQLQ, a pairwise meta-analysis comparing INAH versus OAH included two RCTs in SAR^19^. INAH were associated with an improvement in RQLQ (81% probability of a meaningful difference). Consistent results were observed when INAH and OAH were compared in a NMA (data in the EtD link). For PAR, no RCT were identified directly comparing INAH versus OAH. Indirect comparisons from a NMA indicated that INAH were associated with higher RQLQ improvement compared to OAH. (data in the EtD link).
  - Regarding safety, both a pairwise meta-analysis^19^ and a NMA pointed to a higher risk of adverse events associated with the use of INAH compared to OAH (89% probability of a meaningful difference). For PAR, no RCT were identified directly comparing INAH versus OAH. Indirect comparisons from a NMA indicated that the impact of INAH may range from a small decrease to a moderate increase in the frequency of adverse events compared to OAH (data in the EtD link).
- Resources required, cost-effectiveness and equity: A survey of ARIA experts reported that OAH are more widely available than INAH. In 33 out of the 36 countries with available data, OAH are less expensive than INAH. In addition, based on utilities computed using MASK-air^®^ data, INAH would not be cost-effective in any country. Three OAHs (cetirizine, fexofenadine and loratadine) (but no INAH) are included in the WHO List of Essential Medicines.
- Acceptability and feasibility: MASK-air^®^ data suggest that OAHs are associated with higher treatment satisfaction and lower adherence compared to INAHs^20^. In addition, INAH are more frequently used in co-medication than OAH. The onset of action of INAH and OAH is similar, although favouring INAH.
- Planetary health: No specific evidence was found in terms of comparative impact on planetary health.

**References**

1. Sousa-Pinto B, Vieira RJ, Marques-Cruz M, et al. Artificial Intelligence-Supported Development of Health Guideline Questions. *Ann Intern Med*. 2024;177(11):1518-1529. doi:10.7326/ANNALS-24-00363

2. Guyatt GH, Oxman AD, Kunz R, et al. GRADE guidelines: 2. Framing the question and deciding on important outcomes. *J Clin Epidemiol*. 2011;64(4):395-400. doi:10.1016/j.jclinepi.2010.09.012

3. *GRADEpro GDT: GRADEpro Guideline Development Tool [Software]. McMaster University and Evidence Prime, 2024. Available from gradepro.org.*

4. Alonso-Coello P, Schunemann HJ, Moberg J, et al. GRADE Evidence to Decision (EtD) frameworks: a systematic and transparent approach to making well informed healthcare choices. 1: Introduction. *BMJ*. 2016;353:i2016. doi:10.1136/bmj.i2016

5. Alonso-Coello P, Oxman AD, Moberg J, et al. GRADE Evidence to Decision (EtD) frameworks: a systematic and transparent approach to making well informed healthcare choices. 2: Clinical practice guidelines. *BMJ*. 2016;353:i2089. doi:10.1136/bmj.i2089

6. Neumann I, Anto JM, Bousquet J, Schunemann HJ. The impact of climate change on health needs structured evidence assessment and an evidence to action framework to make decisions: a proposal to adopt the GRADE approach. *J Clin Epidemiol*. 2023;157:146-153. doi:10.1016/j.jclinepi.2023.01.006

7. Vieira RJ, Sousa-Pinto B, Herrmann A, et al. A novel approach to consider Planetary Health in guideline development: a GRADE approach using the Allergic Rhinitis and its Impact on Asthma (ARIA) 2024-2025 guidelines as a case-study. *J Allergy Clin Immunol Pract*. 2025;doi:10.1016/j.jaip.2025.04.060

8. Vieira RJ, Gil-Mata S, Ferreira A, et al. Efficacy and safety of oral antihistamines for allergic rhinitis: Network meta-analysis. *J Allergy Clin Immunol Pract*. 2026.

9. Castillo M, Scott NW, Mustafa MZ, Mustafa MS, Azuara-Blanco A. Topical antihistamines and mast cell stabilisers for treating seasonal and perennial allergic conjunctivitis. *Cochrane Database Syst Rev*. 2015;2015(6):Cd009566. doi:10.1002/14651858.CD009566.pub2

10. Krungkraipetch L, Tansavadi T, Krungkraipetch D. Ranking the efficacy of topical treatments for ocular allergy: A network meta-analysis of current evidence. *Ocul Surf*. 2025;37:273-282. doi:10.1016/j.jtos.2025.05.003

11. Uppsala Monitoring Centre. About VigiBase. <https://who-umc.org/vigibase/>

12. Brozek J, Borowiack E, Sadowska E, et al. Patients' values and preferences for health states in allergic rhinitis-An artificial intelligence supported systematic review. *Allergy*. 2024;79(7):1812-1830. doi:10.1111/all.16100

13. World Health Organization. WHO Model List of Essential Medicines-23rd list. *Geneva: World Health Organization*. 2023;

14. Piggott T, Baldeh T, Dietl B, et al. Standardized wording to improve efficiency and clarity of GRADE EtD frameworks in health guidelines. *J Clin Epidemiol*. 2022;146:106-122. doi:10.1016/j.jclinepi.2022.01.004

15. Food and Drug Administration. *FDA requires Boxed Warning about serious mental health side effects for asthma and allergy drug montelukast (Singulair); advises restricting use for allergic rhinitis*. 2020. *Drug Safety Communication*. <https://www.fda.gov/drugs/drug-safety-and-availability/fda-requires-boxed-warning-about-serious-mental-health-side-effects-asthma-and-allergy-drug>

16. Busby J, Khezrian M, Patel S, Tran TN, Rhodes K, Heaney LG. Exploring the carbon footprint of severe asthma and change after biologic therapy initiation: an analysis of Northern Irish data. *ERJ Open Res*. 2025;11(3)doi:10.1183/23120541.01009-2024

17. Bender BG, Berning S, Dudden R, Milgrom H, Tran ZV. Sedation and performance impairment of diphenhydramine and second-generation antihistamines: a meta-analysis. *J Allergy Clin Immunol*. 2003;111(4):770-6. doi:10.1067/mai.2003.1408

18. Church MK, Maurer M, Simons FE, et al. Risk of first-generation H(1)-antihistamines: a GA(2)LEN position paper. *Allergy*. 2010;65(4):459-66. doi:10.1111/j.1398-9995.2009.02325.x

19. Torres MI, Gil-Mata S, Bognanni A, et al. Intranasal Versus Oral Treatments for Allergic Rhinitis: A Systematic Review With Meta-Analysis. *J Allergy Clin Immunol Pract*. 2024;12(12):3404-3418. doi:10.1016/j.jaip.2024.09.001

20. Sousa-Pinto B, Vieira R, Bognanni A, et al. Comparison of allergic rhinitis treatments on patient satisfaction: A MASK-air® and EAACI Methodological Committee Report. *Allergy*. 2025;doi:<https://doi.org/10.1111/all.70055>
